# Targeting APLN/APJ restores blood-testis barrier and improves spermatogenesis in murine and human diabetic models

**DOI:** 10.1038/s41467-022-34990-3

**Published:** 2022-11-28

**Authors:** Ke Song, Xinyan Yang, Geng An, Xinyu Xia, Jiexiang Zhao, Xiaoheng Xu, Cong Wan, Tianyuan Liu, Yi Zheng, Shaofang Ren, Mei Wang, Gang Chang, Shane J. F. Cronin, Josef M. Penninger, Tao Jing, Xianghong Ou, Shuan Rao, Zhaoting Liu, Xiao-Yang Zhao

**Affiliations:** 1 State Key Laboratory of Organ Failure Research, Department of Developmental Biology, School of Basic Medical Sciences, Southern Medical University, Guangzhou, Guangdong 510515 China; 2 Reproductive Medicine Center of The Third Affiliated Hospital of Guangzhou Medical University, Guangzhou, 510150 China; 3 Department of Bioinformatics, School of Basic Medical Sciences, Southern Medical University, Guangzhou, 510515 China; 4 Guangdong Provincial Key Laboratory of Regional Immunity and Diseases, Department of Biochemistry and Molecular Biology, Shenzhen University Health Science Center, Shenzhen, Guangdong China; 5 IMBA, Institute of Molecular Biotechnology of the Austrian Academy of Sciences, Vienna, Austria; 6 Department of Medical Genetics, Life Sciences Institute, University of British Columbia, Vancouver, BC Canada; 7 Reproductive Medicine Center, Guangdong Second Provincial General Hospital, Guangzhou, 510317 China; 8 Department of Thoracic Surgery, Nanfang Hospital, Southern Medical University, Guangzhou, Guangdong 510515 China; 9 Department of Obstetrics and Gynecology, Zhujiang Hospital, Southern Medical University, Guangzhou, Guangdong China; 10 National Clinical Research Center for Kidney Disease, Guangzhou, China; 11 Experimental Education/Administration Center, School of Basic Medical Science, Southern Medical University, Guangzhou, 510515 China; 12 Key Laboratory of Mental Health of the Ministry of Education, Guangdong-Hong Kong-Macao Greater Bay Area Center for Brain Science and Brain-Inspired Intelligence, Southern Medical University, Guangzhou, Guangdong China

**Keywords:** Type 2 diabetes, Metabolic disorders, Infertility

## Abstract

Type 2 diabetes mellitus is one of the most prevalent metabolic diseases presenting with systemic pathologies, including reproductive disorders in male diabetic patients. However, the molecular mechanisms that contributing to spermatogenesis dysfunction in diabetic patients have not yet been fully elucidated. Here, we perform STRT-seq to examine the transcriptome of diabetic patients’ testes at single-cell resolution including all major cell types of the testis. Intriguingly, whereas spermatogenesis appears largely preserved, the gene expression profiles of Sertoli cells and the blood-testis barrier (BTB) structure are dramatically impaired. Among these deregulate pathways, the Apelin (APLN) peptide/Apelin-receptor (APJ) axis is hyper-activated in diabetic patients’ testes. Mechanistically, APLN is produced locally by Sertoli cells upon high glucose treatment, which subsequently suppress the production of carnitine and repress the expression of cell adhesion genes in Sertoli cells. Together, these effects culminate in BTB structural dysfunction. Finally, using the small molecule APLN receptor antagonist, ML221, we show that blocking APLN/APJ significantly ameliorate the BTB damage and, importantly, improve functional spermatogenesis in diabetic db/db mice. We also translate and validate these findings in cultured human testes. Our findings identify the APLN/APJ axis as a promising therapeutic target to improve reproduction capacity in male diabetic patients.

## Abstract (web summary)

Type 2 diabetes often presents with reproductive disorders, including reduced male and female fertility. Here they show that diabetes disrupts the APLN signaling axis in the testes and that spermatogenesis can be restored through treatment with an APLN antagonist.

## Introduction

Diabetes mellitus (DM) is characterized as a metabolic disorder resulting from deficient insulin secretion, resistance to insulin, or both^1^. Type 2 diabetes mellitus (T2DM) is one of the most prevalent chronic diseases, affecting over 425 million people worldwide^2^. Although T2DM can induce damage in almost all the organs, most studies have focused on the pathogenesis of vital organs such as kidney or retina under high glucose stress^3,4^. However, recently attention has focused on diabetes-associated reproductive dysfunction due to the concerning fact that more individuals are being diagnosed and affected by diabetes in their reproductive ages^5,6^.

Increasing evidence suggests a higher incidence of infertility and spontaneous abortion rates in diabetic population due to dysfunctional spermatogenesis and about 50% of men with diabetes exhibit relative subfertility^7,8^. Although several drugs are available clinically to improve sperm quality and assisted reproductive success for diabetic patients, such as carnitine, glutathione, and vitamin E, their outcomes are not satisfying^9–12^. Although previous studies had reached the consensus that the decline of male fertility was caused by diabetes, most of them were based on clinical data statistical analysis while mechanism interpretation of how hyperglycemia induced subfertility was still lacking^13,14^. Studies from animal models revealed that high glucose-associated damage to spermatogenesis included increased sperm DNA damage, increased testicular oxidative stress, impaired mitochondrial function, hypothalamic pituitary gonadal axis function damage, and a series of factors such as increased advanced glycosylation end products^15–18^. However, whether these findings could be reproduced in human clinical samples remain unclear. Therefore, there are no targeted drugs to directly restore the spermatogenesis in male diabetic patients.

To explore the molecular mechanism of how T2DM impairs spermatogenesis, it is important to investigate cell-type-specific gene expression differences between diabetic patients and healthy controls. Previously, it was a major challenge to obtain the transcriptome data of the entire testis at one time as only a few specific cell subpopulations could be obtained using traditional flow cytometry sorting method which became even more difficult to investigate when external conditions changed^19^. The development of single-cell RNA-sequencing technology allowed us to determine sequential cell fate transitions throughout human spermatogenesis^20,21^.

APLN is a peptide which has several isoforms including Apelin-13, Apelin-17, and Apelin-36. Interestingly, Apelin −13 is the shortest form of APLN but with the highest biological activity^22,23^. APLN and its specific receptor APJ are wildly expressed in the heart, hypothalamus-pituitary axis, ovary, and many other organs^22–26^. Importantly, it is demonstrated that APLN is highly expressed in both T2DM patients and diabetic mice^27^, and that the APLN/APJ axis is an important regulator in the diabetes development and diabetic complications^28^. Recently, there are increasing evidences showing that APLN/APJ plays a role in regulating male fertility and diabetes-associated infertility. For instance, it has been shown that Apelin-13 can reduce testosterone release by inhibiting the secretion of luteinizing hormone;^29^ another study shows that APLN expression is increased in the testes of the T1DM mouse model, and testes cultured with ML221 significantly increased testosterone secretion^30^, however, whether APLN/APJ is involved in diabetic-associated spermatogenesis dysfunction remains unclear.

Here, we reported an over-activation of APLN/APJ axis in T2DM patients’ testis using single-cell RNA sequencing technology, we aim to use both *in* and ex vivo systems to explore the role of APLN/APJ in impairing normal spermatogenesis under hyperglycemic conditions, and whether targeting APLN/APJ serves as a promising strategy to improve spermatogenesis for diabetic patients.

## Results

### Single-cell transcriptome profiling of testicular cells in T2DM

**Fig. 1:**
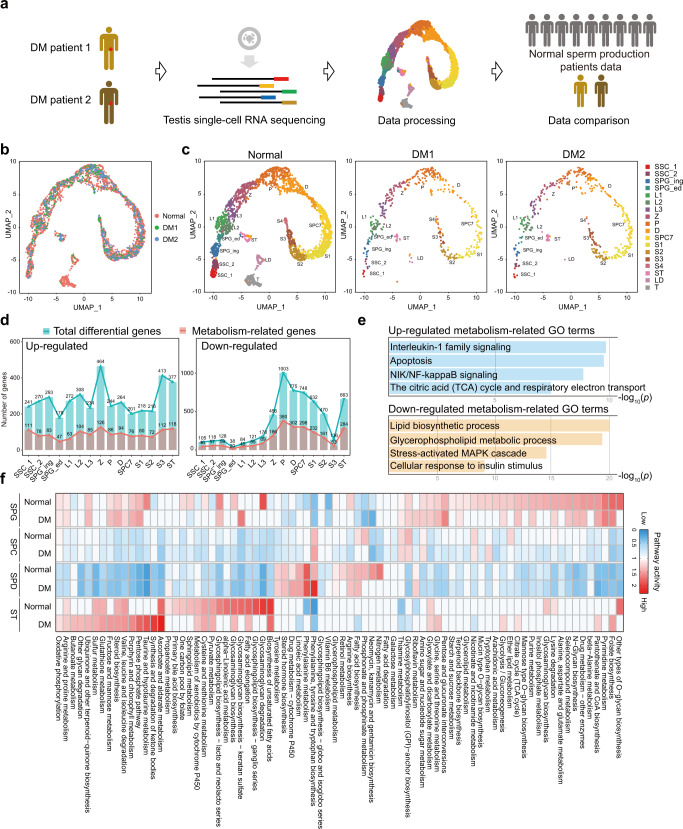
Single-cell transcriptome atlas of the human testis in health and T2DM. **a** Schematic illustration of the experimental and analysis workflow. **b** UMAP and clustering analysis of single-cell transcriptome data from normal spermatogenesis human testis (*n* = 2810), integrated with single-cell transcriptome data of two Type 2 diabetes mellitus (T2DM)’ testes (DM patient 1 (DM1), *n* = 441; DM patient 2 (DM2), *n* = 459). Each dot represents a single cell and is colored according to its donor of origin. **c** UMAP plots showing the cell colors by the identified cell clusters between normal and diabetic patients. **d** Histogram showing the number of (left) upregulated differential expression genes and (right) downregulated differential genes from the normal and diabetic patients single-cell RNA sequencing data. The blue histogram represented total differential genes, and the red represented metabolic genes. **e** Enriched GO terms in the upregulated and downregulated metabolism-related genes of testicular cells from diabetic versus normal. The statistical test is a hypergeometric test. **f** Metabolic pathway activities in the testicular cell types including spermatogonia (SPG), spermatocyte (SPC), spermatid (SPD), and Sertoli (ST) cells from the normal and diabetic single-cell RNA sequencing data.

To comprehensively analyze the transcriptome variation of testicular cells from T2DM patients, we obtained intact seminiferous epithelium from two T2DM patients via testicular sperm aspiration (TESA) with ethics approval (Fig. 1a). We used random cell picking (unsorted) strategy for sample collection to capture all the spermatogenic cell types. Moreover, to capture more transcripts compared to 10× genomic sequencing, STRT-seq method (based on SMART-seq2) was used to achieve deeper sequencing for further analysis. After strict quality control, a total of 900 single-cell transcriptomes were retained for the subsequent analysis (Supplementary Fig. 1a). Next, a previous reported single-cell RNA sequencing (scRNA-seq) data from 2810 normal testicular cells of nine persons was re-analyzed which used the same strategy as the current study^31^. The Dimension reduction and clustering analysis was then performed for cell type identification^32^. Not surprisingly, the clustering results showed that diabetic patients’ testicular cells were well distributed compared with normal controls (Fig. 1b). Particularly, normal and diabetic patients’ testicular cells were organized as a continuous arc of 15 clusters as well as another 3 separate clusters (Fig. 1c), and the distribution of selected cells in each spermatogenesis stage was comparable between normal and diabetic patients (Supplementary Fig. 1b, c). We also observed similar marker gene expression for germ cells (e.g., *DDX4*, *GFRA1*, and *SYCP1*) and Sertoli cells (*AMH*) as well as Leydig cells (*MYH11*) between healthy and diabetic testicular cells (Supplementary Fig. 1d, e). A pseudotime analysis using the Monocle package^33^ further revealed that the healthy and diabetic patients shared same pseudotime trajectory (Supplementary Fig. 1f). Finally, immunostaining was performed to validate the expression of classical marker genes throughout the spermatogenic cell type, which also indicated that there was no obvious difference between T2DM and normal control (Supplementary Fig. 1g–l).

The process of spermatogenesis in T2DM patients could be further interpreted by analyzing differentially expressed genes (DEGs) as identified by scRNA-seq. Intriguingly, nearly half of DEGs at each stage were metabolism-relevant (Fig. 1d and Supplementary Data 1). Gene ontology (GO) analysis of metabolism-related genes suggested that upregulated genes in diabetic patients’ testes were enriched in “Apoptosis”, “NF-κB signaling”, “Interleukin-1 family signaling” as well as “The citric acid (TCA) cycle, and respiratory electron transport”, whereas genes annotated to “Lipid biosynthetic process”, “Glycerophospholipid metabolic process”, “Stress-activated MAPK cascade” and “Cellular response to insulin stimulus” were downregulated in T2DM compared to normal (Fig. 1e). As diabetes is a metabolic disorder, we next investigated which spermatogenic stage was most affected by hyperglycemia. To this end, we adopted a pipeline for analyzing metabolic gene expression profiles to explore the metabolic pathway variation across all cell types^34^ (Supplementary Fig. 2a). We then divided all the sequenced cells according to the meiotic stage, including spermatogonia (SPG, SSC_1 to SPG_ed), spermatocyte (SPC, L1 to SPC7), spermatid (SPD, S1 to S4), and Sertoli (ST) cells (Fig. 1f), which showed that metabolic pathways were dramatically changed in SPG and Sertoli cells (Fig. 1f). The top20 up- and downregulated metabolic pathways in SPG, SPC and SPD stages were outlined respectively (Supplementary Fig. 2b), we found citrate cycle was abnormally elevated in the spermatogonia stage in diabetic patients (Supplementary Fig. 2c), which might lead to impair the pluripotency of spermatogonial stem cells in diabetic patients^35^ as we observed (Supplementary Fig. 2d–f). In this study, we are particularly interested with those potential influences of Sertoli cells dysfunction on spermatogenesis, as the activity of several metabolic pathways was significantly changed in Sertoli cells of T2DM patients (Fig. 1f).

### Defective metabolism in ST contributes to dysfunctional BTB

**Fig. 2:**
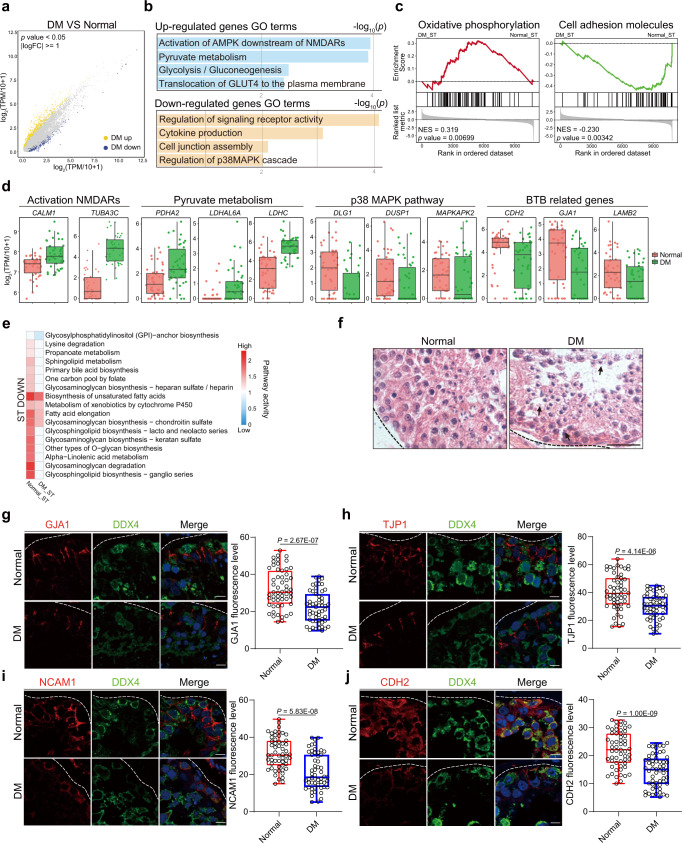
Single-cell analysis of human diabetic testis suggests impaired cell junction in BTB. **a** Scatter plot showing the DEGs in Sertoli cells between diabetic patients and normal spermatogenesis. **b** Enriched GO terms in the upregulated and downregulated genes of Sertoli cells from diabetic versus with normal control. The statistical test is a hypergeometric test. **c** GSEA analysis showing the upregulated and downregulated pathway activity in Sertoli cells. **d** Boxplots showing the expression levels of Activation NMDARS, Pyruvate metabolism, P38 MAPK pathway, BTB related genes (Normal = 43 cells, DM = 42 cells). Each box represents the median and the 25% and 75% quartiles, and the whiskers indicate 1.5 times of the interquartile range. **e** Heatmap showing the representative decreased metabolic pathway activity from the normal control and diabetic data in Sertoli cells. **f** H&E staining of adult human testicular paraffin sections from donors with normal control (left) and diabetic (right), arrow indicated vacuoles and free cells in seminiferous tubules. Scale bar, 20 μm. **g**–**j** Immunofluorescence of GJA1, TJP1, NACM1 and CDH2 (red) co-stained with DDX4 (green) in normal and diabetic patients’ testicular paraffin sections. *n* = 3 testis samples per group. Representative immunofluorescence images and quantitative analysis of GJA1, TJP1, NACM1 and CDH2. Scale bar, 10 μm. Representative immunofluorescence images and quantitative analysis of CDH2. Scale bar, 10 μm. Fluorescence intensity values of more than 50 positive cells in at least 5 fields of view were counted in normal and diabetic patients’ testicular paraffin sections. Two-tailed student’s t test was performed. Box-and-whisker plots denote the maximum (top whisker), 75th (top edge of box), 25th (bottom edge of box), and minimum (bottom whisker) percentiles, and the median (line in box).

It is well established that the critical roles of Sertoli cells are to form the BTB and provide nutrients for proper spermatogenesis^36^. Indeed, DEGs analysis results from scRNA-seq date provided clues for an aberrant Sertoli cell function in T2DM patients (Fig. 2a and Supplementary Data 2). In line with previous findings, our data suggested that most upregulated genes were enriched in “Translocation of GLUT4 to the plasma membrane”, “Activation of AMPK downstream of (NMDA receptor) NMDAR”, “Pyruvate metabolism” and “Glycolysis/Gluconeogenesis^37–40^; in contrast, the majority of downregulated genes were annotated in “Regulation of signaling receptor activity”, “Cytokine production”, “Regulation of p38MAPK cascade” and “Cell junction assembly” (Fig. 2b). Gene Set Enrichment Analysis (GSEA) showed that oxidative phosphorylation and cell adhesion molecules (CAMs) were prominently changed in Sertoli cells of T2DM patients (Fig. 2c, d and Supplementary Data 2), consistent with the finding that glycosaminoglycans synthesis in Sertoli cells were suppressed under diabetic conditions (Figs. 1f and 2e). These results indicated that the metabolic homeostasis in Sertoli cells is disrupted under diabetic conditions, which might further lead to the structural or functional impairment of BTB in T2DM patients. H&E staining showed that the germ cells were not properly organized in the seminiferous epithelium, wherein there were many vacuoles and free cells in the center relative to normal control (Fig. 2f). In addition, the expression of tight junction markers TJP1^41^ and GJA1^42^, as well as the adherent junction markers CDH2^43^ and NCAM1^44^ was significantly downregulated in diabetic testis (Fig. 2g–j). Together, these results suggested that the BTB damage was occurred in T2DM patient’s testis.

To further dissect how diabetic conditions would influence the integrity of BTB and spermatogenesis, further experiments were performed on the widely used diabetic mouse model. The combination of streptozotocin and high-fat diet is a classical murine hyperglycemia model (HGM) to recapitulate human diabetes development^45^ (Supplementary Fig. 3a). The parameters including body weight, fasting glucose, islet atrophy and insulin secretion confirmed the diabetic phenotypes (Supplementary Fig. 3b–e). Notably, we also found a decrease in the thickness of the seminiferous tubules and a significant decrease in the number of germ cells in the seminiferous epithelium (Supplementary Fig. 3f). Importantly, a similar phenotype was reproduced in HGM testis as in diabetic patient’s testis (Supplementary Fig. 3g). In vivo biotin tracing assay is a direct method to evaluate BTB integrity and permeability^46^. Using this method, we found a significant augmentation of biotin-positive seminiferous tubules and a marked increase in biotin penetration distance in the testis of HGM mice, indicating a compromised BTB integrity in HGM mice (Supplementary Fig. 3h, i). Similarly, immunostaining results showed that the cell junction markers were significantly decrease in the Sertoli cells of diabetic mice (Supplementary Fig. 3j–m). To further investigate whether defective BTB would impact the quality of germ cells, intracytoplasmic sperm injection (ICSI) was performed using the spermatozoa of HGM mice. We found a significantly decreased blastocyst rate (33%) in HGM mice compared to control mice (52.7%) (Supplementary Fig. 4a–c). Together, these results indicate that hyperglycemia disrupts the metabolic homeostasis of Sertoli cells, which further results in the damage of BTB and ultimate dysfunctional spermatogenesis in both diabetic patients and mice.

### Excessive APLN perturbs the integrity and function of BTB

**Fig. 3:**
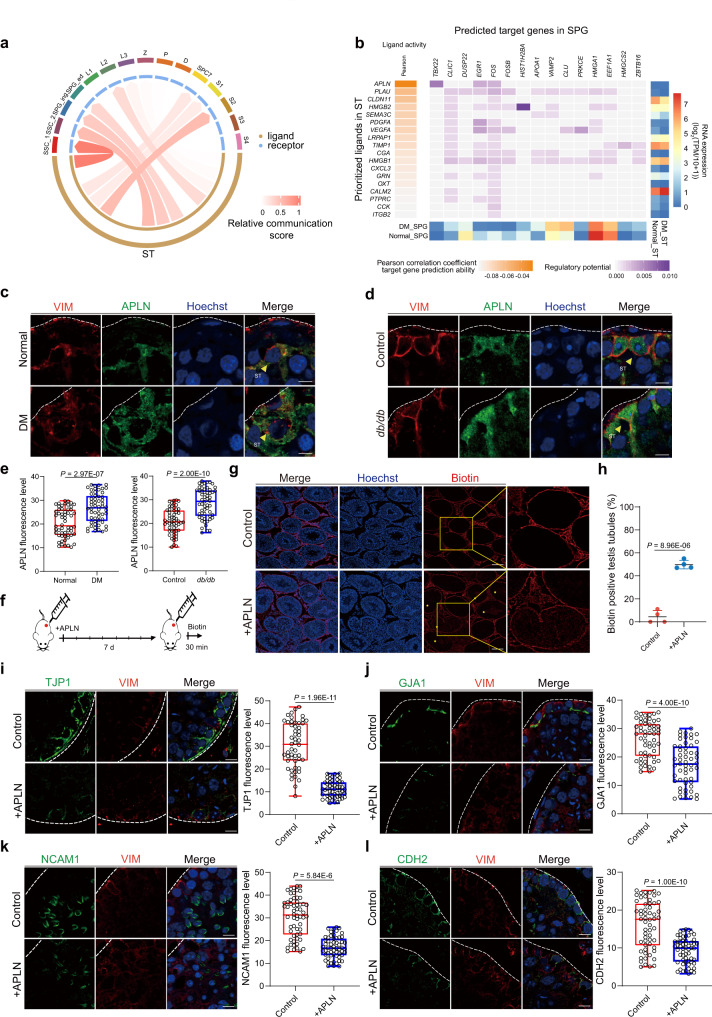
Excess APLN induces BTB dysfunction. **a** Chord diagram showing the relationship and strength of the regulatory network between Sertoli cells and germ cells. **b** Heatmap showing the relationship between ligands expressed by Sertoli cells and potential target genes in spermatogonia (SPG) cells. A gradient of gray, orange indicates low to high Pearson correlation coefficient target gene prediction ability. And, a gradient of gray, purple indicates low to high ligand-target regulatory potential. The expression of the corresponding ligands and target genes are displayed on the right and below in the form of heatmaps. **c** Immunofluorescence of VIM (red) co-stained with APLN (green) in normal and diabetic patients’ testicular paraffin sections. Scale bar, 10 μm. The yellow arrow indicates the Sertoli cells. **d** Immunofluorescence of VIM (red) co-stained with APLN (green) in control and *db/db* testicular paraffin sections. Scale bar, 10 μm. The yellow arrow indicates the Sertoli cells. **e** Quantitative analysis of APLN. Box-and-whisker plots denote the maximum (top whisker), 75th (top edge of box), 25th (bottom edge of box) and minimum (bottom whisker) percentiles, and the median (line in box). *n* = 3 testis samples per group. Two-tailed student’s t test was performed. **f** Schematic illustration of the APLN testicular injection experiment in C57BL/6N. **g** Biotin tracer was injected in the C57BL/6N interstitium of the testicular tissue. Cy3-conjugated streptavidin was used to detect the presence of biotin in the adluminal compartment in control and APLN injection mouse testicular paraffin sections. Scale bar, 50 μm. **h** Biotin positive seminiferous tubules percentage in control and APLN injection group. Data are presented as means ± SEM. Unpaired two-tailed t test. Statistics were performed in four mouse testes each group (*n* = 4). **i**–**l** Immunofluorescence of VIM (red) co-stained with TJP1, GJA1, NCAM1, and CDH2 (green) in control and APLN injection mouse testicular paraffin sections. *n* = 3 biologically independent mice per group. Representative immunofluorescence images and quantitative analysis of TJP1, GJA1, NCAM1, and CDH2. Scale bar, 10 μm. Two-tailed student’s t test was performed. Box-and-whisker plots denote the maximum (top whisker), 75th (top edge of box), 25th (bottom edge of box) and minimum (bottom whisker) percentiles, and the median (line in box).

The BTB constituted predominantly by the Sertoli cells forms an isolated microenvironment for spermatogenesis. Moreover, BTB also serves as a signaling hub to relay external signals which are essential for appropriate spermatogenesis^47^. As aforementioned, the activity of many metabolic pathways in Sertoli cells were severely affected and most DEGs associated with signaling receptor activity were downregulated in T2DM patients. Therefore, we optimized a previously reported algorithm to overview the ligand/receptor network between Sertoli cells and other germ cells^48^ (Fig. 3a), which revealed that most receptors/ligands interactions existed between Sertoli cells and spermatogonia. Next, we performed ligand activity prediction to find the deregulated ligands in Sertoli cells and their downstream targeted genes in SPG, which highlighted APLN as the top candidate involved in dysfunctional Sertoli cells under diabetic conditions^49^ (Fig. 3b). Indeed, APLN protein expression was highly upregulated in human diabetic testis, particularly in Sertoli cells compared to the normal control (Fig. 3c). The upregulation of APLN could also be observed in *db/db* mice, another classical diabetic mouse model^50^ (Fig. 3d, e). However, the expression of APJ (APLN receptor) was not changed in diabetic human or mice (Supplementary Fig. 5a–c).

To assess the direct effect of excessive APLN to BTB, we injected APLN peptide into the testicular interstitium of normal mice for 7 days (Fig. 3f) and then performed biotin tracer assay to assess the integrity of BTB. Similar to HGM mice, we observed a significant increase of biotin-positive seminiferous tubules in mice treated with APLN injection (Fig. 3g, h), suggesting that excessive APLN treatment alone could damage the BTB structure. This finding was further validated by decreased immunostaining signals for tight junction (TJP1, NCAM1), gap junction (GJA1), and basal ectoplasmic specializations (CDH2) (Fig. 3i–l) in Sertoli cells of mice treated with APLN injection. These results suggest that the abnormal upregulation of APLN in diabetic patients leads to defective spermatogenesis by impairing BTB-associated proteins.

### High glucose induces APLN expression via HIF1A activation

**Fig. 4:**
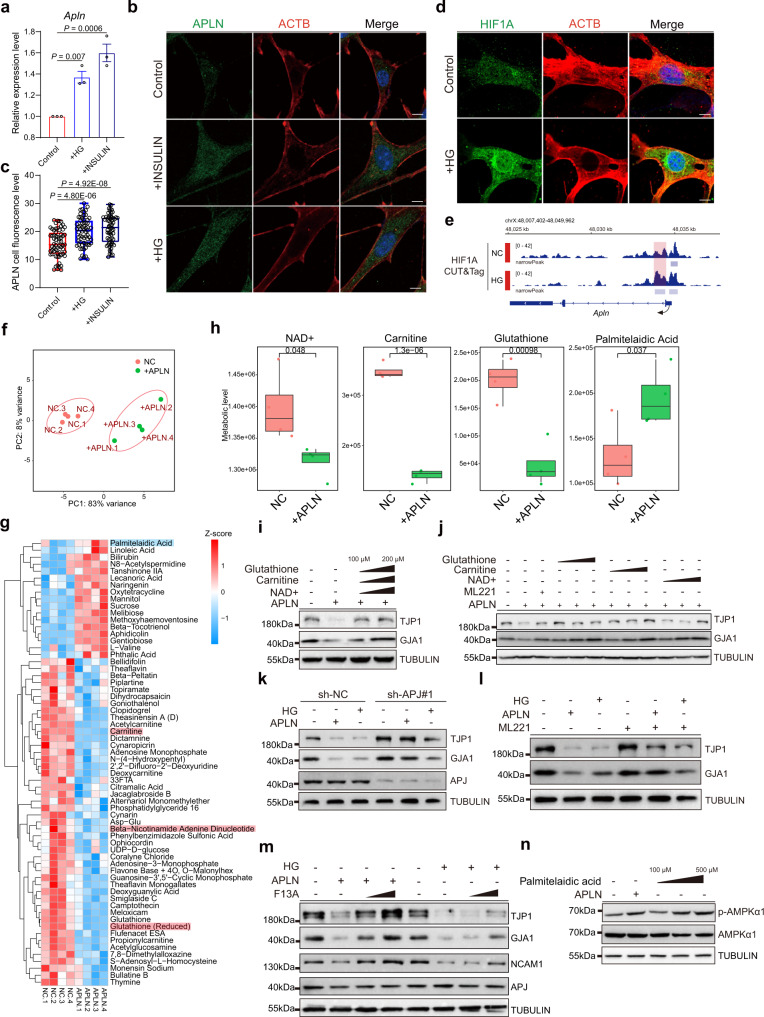
High glucose-associated APLN upregulation impairs metabolic homeostasis in TM4 Sertoli cell. **a** The expression level of *Apln* mRNA in TM4 cell under high glucose (HG) and insulin. Data are presented as means ± SEM. Unpaired two-tailed t test. Results of three independent experiments are shown (*n* = 3). **b** Immunofluorescence of ACTB (red) co-stained with APLN (green) in TM4 cell under HG and insulin. Scale bar, 5 μm. **c** Quantitative analysis of APLN immunofluorescence level in (**b**). Box-and-whisker plots denote the maximum (top whisker), 75th (top edge of box), 25th (bottom edge of box) and minimum (bottom whisker) percentiles, and the median (line in box). Unpaired two-tailed t test. *n* = 100 cells examined over 3 independent experiments. **d** Immunofluorescence of ACTB (red) co-stained with HIF1A (green) in TM4 cell under HG. Scale bar, 5 μm. **e** Genome browser view of the HIF1A density in the *Apln* range in negative control (NC) and HG treated TM4 cell. **f** PCA analysis of metabolomic data in NC and APLN group. Four biological replicates were performed for each group. **g** Heatmap showing all differential metabolites in NC and APLN group. **h** The metabolic level of NAD+, carnitine and glutathione and Palmitelaidic Acid in NC and APLN group. unpaired two-tailed t test between sample groups. *n* = 4 biologically independent samples. Each box represents the median and the 25 and 75% quartiles, and the whiskers indicate 1.5 times of the interquartile range. **i**, Immunoblots of TJP1 and GJA1 in TM4 treated APLN and by combination of three metabolites in different concentrations. **j** Immunoblots of TJP1 and GJA1 in TM4 treated APLN and three different concentrations (200, 500, 1000 μM) of each metabolites separately. **k** Immunoblots of indicated proteins in stable knockdown APJ cell treated APLN or HG. **l** Immunoblots of indicated proteins in TM4 treated APLN or HG and rescued by ML221. **m** Immunoblots of indicated proteins in TM4 treated APLN or HG and rescued by F13A. **n** Immunoblots of AMPKα1 and p-AMPKα1 in TM4 treated APLN or different concentrations of Palmitelaidic Acid.

To directly define the link between high glucose and APLN expression, we adapted a mouse Sertoli cell line TM4^51^ to mimic the microenvironment of BTB under hyperglycemia conditions. We found that treatment by high glucose or insulin treatment could significantly induce the expression of APLN in TM4 cells (Fig. 4a–c). Mechanistically, we showed that high glucose induced an obvious reactive oxygen species (ROS) production and HIF1A activation in TM4 cells which could be blocked by HIF1A inhibitor (Supplementary Fig. 6a–d), moreover, HIF1A could translocate into nucleus and subsequently bind to APLN promoter to trigger APLN expression upon high glucose treatment (Fig. 4d, e).

We then assessed whether high APLN treatment could impair cell adhesion in Sertoli cells as we observed in vivo. Interestingly, treatment with either native APLN or APJ agonists, Apelin-13 and Pyr1-Apelin-13, had no impact on cell identity (Supplementary Fig. 7a), proliferation (Supplementary Fig. 7b, c) and cell death (Supplementary Fig. 7d, e). However, for the cell junction protein, their expression was significantly lower in TM4 cells (Supplementary Fig. 7f–k). To examine whether cell adhesion was changed, we performed cell adhesion assays on APLN-treated cells (Supplementary Fig. 8a) and found that APLN-treated cells showed a significant reduction in cell-cell adhesion (Supplementary Fig. 8b–d).

### Activation of APLN/APJ disrupts metabolic homeostasis

Since diabetes-associated male reproductive dysfunction is a metabolic disorder, we asked whether activation of APLN/APJ had any effect on the metabolism of Sertoli cells. Using metabolomic profiling, we demonstrated that APLN treatment induced a clear metabolic change in Sertoli cells (Fig. 4f, g). In particular, several well-recognized reproductive associated metabolites, such as β-Nicotinamide Adenine Dinucleotide (NAD+), carnitine and glutathione^52^ were significantly downregulated in APLN treated Sertoli cells, whereas Palmitelaidic Acid was clearly induced upon APLN treatment (Fig. 4h).

We have demonstrated that APLN treatment dramatically suppresses the expression of major cell adhesion proteins, thus impairing BTB integrity. We next assessed whether these genes’ expression could be restored using multiple strategies. We showed that adding combinations of downregulated metabolites including NAD+, carnitine and glutathione could efficiently re-establish the expression of the cell junction markers TJP1 and GJA1 (Fig. 4i); importantly, adding NAD+, carnitine, and glutathione individually also inhibited APLN’s effect on TJP1 and GJA1’s expression, which was comparable to incubation of TM4 cells with ML221^53^, a classical APJ small molecular inhibitor (Fig. 4j).

As high glucose and APLN treatment showed similar effect to cell adhesions of TM4 cells (Supplementary Fig. 9a), we then intended to explore if targeting APLN/APJ axis could ameliorate such effect. We first tested whether blocking APLN/APJ using small interfering RNAs targeting APJ could neutralize APLN effect. APJ was successfully knocked down in TM4 cells without affecting cell viability and identity, and had no effect on cell adhesion markers’ expression (Supplementary Fig. 9b–f). However, inhibition of APJ could significantly restore TJP1, GJA1, and NCAM1 expression in TM4 cells with high glucose APLN treatment (Fig. 4k and Supplementary Fig. 9g), not surprisingly, using ML221 or dominant negative form of APLN F13A^54^ (QRPRLSHKGPMPA) also achieved similar outcome as knocking down APJ (Fig. 4l, m).

Previous studies have shown that AMPK and MAPK pathways are two major downstream effectors that are regulated by APLN^55^, we then evaluated whether high glucose or APLN treatment could affect Sertoli cells similarly. Indeed, either high glucose or APLN treatment, or combination of both dramatically activated phosph-AMPKα1 expression whereas suppressed MAPK1/3 activity, all these effects could be efficiently blocked by inoculating TM4 cells with ML221 (Supplementary Fig. 10a). We then treated TM4 cells with MAPK inhibitor Ulixertinib or AMPK agonist AICAR or both, these treatments all significantly suppressed cell adhesion proteins expression in line with high glucose or APLN treatment (Supplementary Fig. 10b, c). Interestingly, one upregulated metabolite recognized by the metabolomics study, Palmitelaidic Acid, could also induce phosph-AMPKα1 expression as an AMPK agonist (Fig. 4n), which consistently corporate the metabolic change and signal transduction upon APLN treatment.

### ML221 restores BTB integrity and improves spermatogenesis

**Fig. 5:**
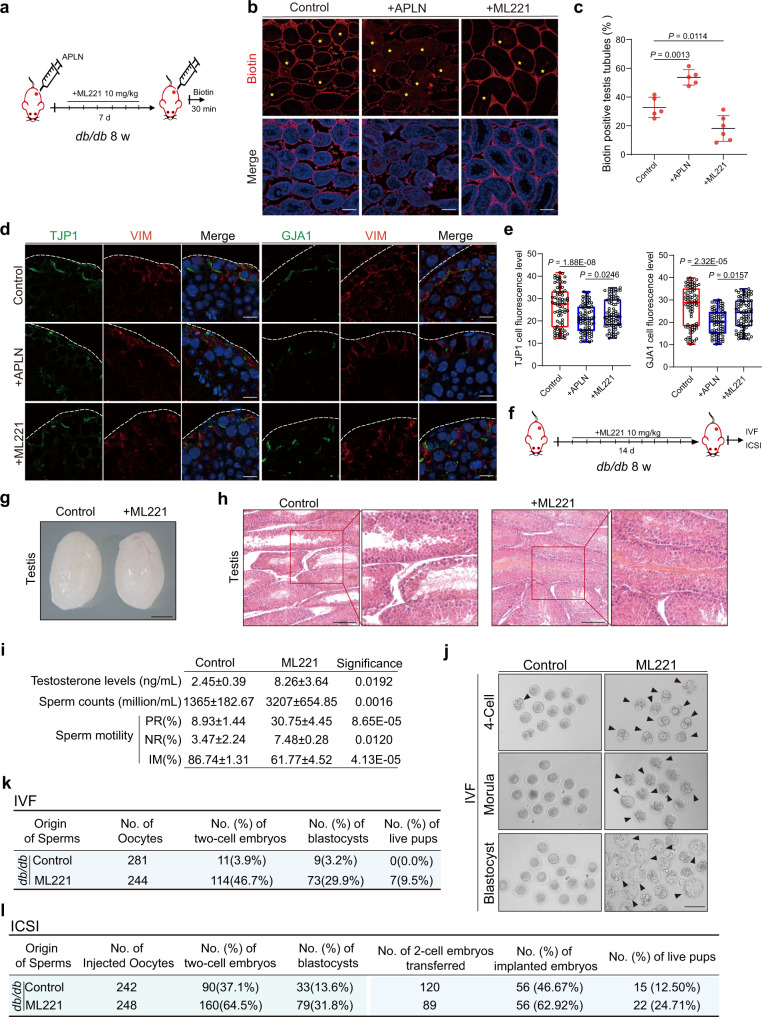
Targeting APLN/APJ repairs BTB damage and improves sperm quality in diabetic mice. **a** Schematic illustration of the APLN or ML221 injection experiment in *db/db*. **b** Immunofluorescence of biotin (red) between indicated sample groups. Scale bar, 50 μm. **c** Biotin positive seminiferous tubules percentage in Control, APLN and ML221 injection group. Data are presented as means ± SEM. One-way ANOVA. Statistics were performed in five mouse testes each group (*n* = 5). **d** Immunofluorescence of TJP1 and GJA1 (green) co-stained with VIM (red) in APLN injection and ML221 injection testicular paraffin sections. Scale bar, 10 μm. **e** Quantitative analysis of TJP1 and GJA1. Box-and-whisker plots denote the maximum (top whisker), 75th (top edge of box), 25th (bottom edge of box) and minimum (bottom whisker) percentiles, and the median (line in box). Statistics were performed in five mouse testes each group (*n* = 5). One-way ANOVA was performed. **f** Schematic illustration of the ML221 injection experiment in *db/db* for IVF and ICSI. Mice were treated with ML221 at a dose of 10 mg per kg body weight per day. **g** Bright field diagram of testicular size in control and ML221 injection group. Scale bar, 2 mm. **h** H&E staining of testicular sections in control and ML221 injection group. Scale bar, 100 μm. **i** Sperm counts, sperm motility and testosterone level between control and ML221 injection group. PR: progressive motile, NP: non-progressive motile, IM: immotility. unpaired two-tailed t test was performed. **j** Brightfield diagram of 4-cell, morula, and blastocyst between control and ML221 injection group. Arrows indicated normal developing embryos. Scale bar, 200 μm. **k** The trilinear table shows all the embryo injection, two-cell embryos, blastocysts and live C-section-born mice data of IVF between control and ML221 injection group. **l** The trilinear table shows all the embryo injection, two-cell embryos, blastocysts and live C-section-born mice data of ICSI between control and ML221 injection group.

As aforementioned that ML221 could efficiently ameliorate APLN’s effect on Sertoli cells, we wondered whether these findings could be expanded in diabetic mice in vivo. We then adapted a classical diabetic *db/db* mouse model to validate our in vitro observations by injecting APLN or ML221 (Fig. 5a). The biotin tracer assay suggested APLN injection in testis severely aggravated BTB damage in *db/db* mice, but continuously ML221 treatment significantly improved BTB integrity in diabetic testis (Fig. 5b, c), which was further confirmed by immunostaining assay with cell adhesion markers (Fig. 5d, e) as well as recapitulated in vitro cultured TM4 cells (Supplementary Fig 11a, b).

In order to as much as possible cover an intact mouse spermatogenesis cycle, we prolonged the duration of ML221 injection to two weeks We performed the experiment to evaluate the effect of ML221 treatment on *db/db* male mating ability directly. The *db/db* male mice were treated with either ML221 or vehicle (*n* = 6 for each group) for two weeks and then mated with estrous female mice (Supplementary Fig. 12a). The results showed that no vaginal (copulatory) plug were found in neither group, suggesting ML221 treatment did not restore natural fertility in *db/db* mice (Supplementary Fig. 12b). Therefore, we used in vitro fertilization (IVF) and ICSI to explore if ML221 could improve sperm quality (Fig. 5f). Long term ML221 treatment had no effect on the size of diabetic testis, kidney and liver (Fig. 5g and Supplementary Fig. 13a, c, e) but substantially promoted the tight arrangement of germ cells in the lumen (Fig. 5h and Supplementary Fig. 13b). There are no significant changes in liver and kidney after long term ML221 treatment (Supplementary Fig. 13d, f). Of note, blood testosterone levels, sperm concentration and motility were both significantly improved in ML221-treated diabetic mice (Fig. 5i) and sperm concentration as well as sperm motility from ML221 treated diabetic mice had restored as comparable to those from wild-type C57BL/6N mouse (Supplementary Fig. 14a–c). More importantly, sperm from ML221-treated diabetic mouse displayed enhanced capacity to fertilize oocytes and support intact embryo development compared with sperm from untreated diabetic mouse by IVF, showing as the elevated developmental rates of 2-cell embryos and blastocysts(46.7 and 29.9% in M221 treated group versus 3.9 and 3.2% in the control group) (Fig. 5j, k). Accordingly, 9.5 and 0.0% of blastocytes derived from M221 and control groups could be developed to full-term, respectively (Fig. 5k) and similar results could also be obtained by ICSI (Fig. 5l and Supplementary Fig. 14d–f). These results indicate that blocking APLN/APJ using ML221 not only restores the BTB integrity and testis structure, but also functionally improves the sperm quality in *db/db* mice.

### ML221 re-establishes cell junction protein expression

**Fig. 6:**
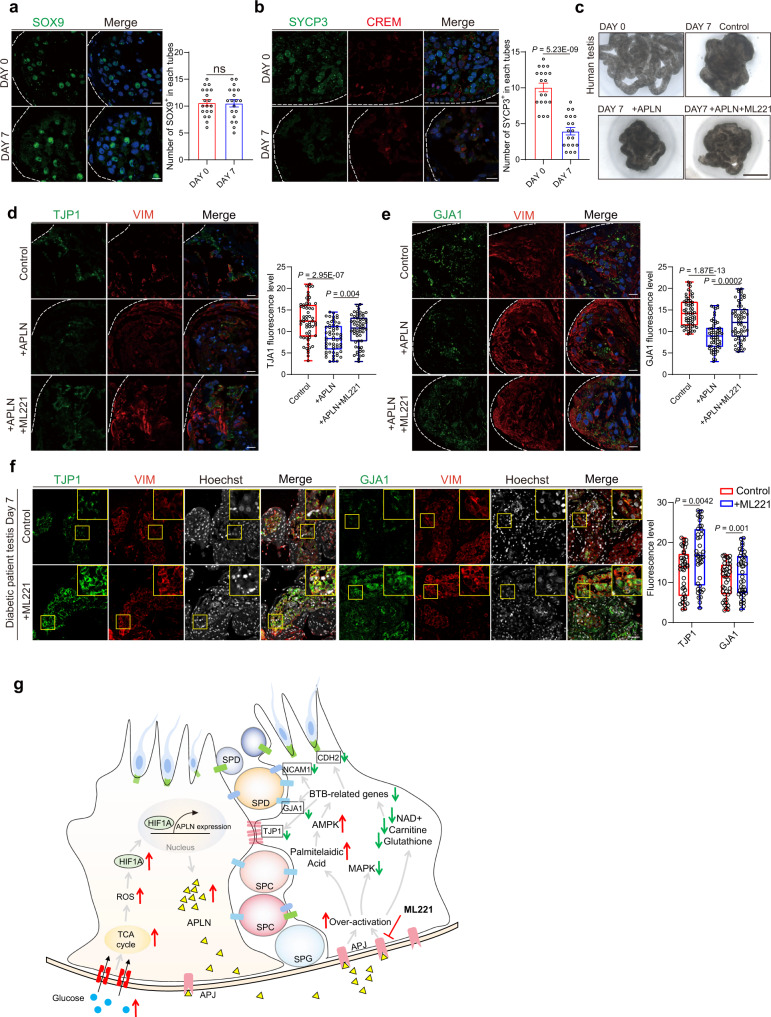
Inhibition APLN improves cell junction protein expression in human cultured testis. **a** Immunofluorescence of SOX9 (red) in human testis culture in Day 0 and Day 7 paraffin sections. Scale bar, 20 μm. The percentage of SOX9-positive cells was calculted on Day 0 and Day 7 separately. Mean ± SEM. ns, not significant, unpaired two-tailed t test. *n* = 3 human testis culture examined over 3 independent experiments**. b** Immunofluorescence of SYCP3 (green) and CREM (red) in human testis culture on Day 0 and Day 7 paraffin sections. Scale bar, 20 μm. The percentage of SYCP3-positive cells was counted. Mean ± SEM. Unpaired two-tailed t test. *n* = 3 human testis culture examined over 3 independent experiments**. c** Bright field diagram of human testis culture between different groups. Scale bar, 50 mm. **d**, **e** Immunofluorescence of TJP1 and GJA1 (green) and VIM (red) in human testis culture in Day 7 paraffin sections between indicated sample groups. *n* = 3 per group. Scale bar, 20 μm. Box-and-whisker plots denote the maximum (top whisker), 75th (top edge of box), 25th (bottom edge of box), and minimum (bottom whisker) percentiles, and the median (line in box). Quantitative analysis of TJP1. Two-tailed student’s t test was performed. **f** Immunofluorescence of TJP1 or GJA1 (green) and VIM (red) in diabetic patient testis culture in Day 7 paraffin sections between indicated sample groups. Scale bar, 50 μm. *n* = 3 per group. Box-and-whisker plots denote the maximum (top whisker), 75th (top edge of box), 25th (bottom edge of box) and minimum (bottom whisker) percentiles, and the median (line in box). Quantitative analysis of TJP1 and GJA1. Two-tailed student’s t test was performed. **g** Hypothetical Mechanism. Elevated blood glucose in diabetic patients directly leads to elevated ROS in Sertoli cells, which promotes HIF1A nuclear translocation and activates *Apln* expression. The excess of APLN disrupted the BTB-related genes by decreasing NAD+, carnitine, and glutathione. Blocking APLN/APJ with F13A and ML221 could significantly ameliorate the BTB damage and improve low sperm quality.

To expand our findings within murine diabetic models, we optimized a temporary human testis culture method^56^ which could maintain Sertoli cell’s identity and activity in a week (Fig. 6a) but lose spermatocytes and round spermatids gradually (Fig. 6b). APLN or ML221 treatment for seven days had no obvious effect on the morphology of cultured human testis (Fig. 6c), however, consistent with our previous findings, we demonstrated that ML221 could efficiently restore the expression of cell adhesion markers including TJP1, CDH2, GJA1 and NCAM1 in human testis, which were rigorously suppressed by APLN treatment (Fig. 6d, e and Supplementary Fig. 15a, b), suggesting targeting APLN/APJ could re-establish the cell junction protein expression, thereby alleviate the BTB damage induced by high glucose or high APLN in diabetic patients (Fig. 6g).

## Discussion

It is well defined that APLN serves as an important regulator in adipogenesis in pro-adipocytes^57^ and stimulates nitric oxide (NO) production to achieve vasodilatation as well as blood pressure reduction^58^. APLN is wildly expressed in many human tissues, whereas the upregulation of APLN is usually observed in the context of obesity and diabetes^27^. Indeed, diabetes can cause multiple sexual dysfunctions such as decreased sperm quality^59^ and erectile abnormality^60^, and thus greatly impair male fertility. Although various studies have shown that diabetes is a risk factor to cause male infertility, the underling molecular mechanism which links hyperglycemia and abnormal spermatogenesis is not fully understood. In the present study, we performed single-cell RNA sequencing of over 900 individual testicular cells collected from diabetic patients, which revealed that over activation of the APLN pathway in diabetic patients was a key signal that mediated BTB dysfunction and sperm quality decline. Thus, our findings provide a direct proof connecting diabetes to male subfertility.

Human testicular germ cells are highly heterogeneous and contain 18 different cell subtypes throughout spermatogenesis^31^. The BTB belongs to the seminiferous epithelium, and is mainly composed of Sertoli cells to sense external signals. One major role of Sertoli cells is to maintain a functional BTB, which constitutes the unique isolated physiological environment required for germ cell development^61^. Using both in and ex vivo models, we demonstrated that high level glucose could increase APLN expression in Sertoli cells, which subsequently inhibited the expression of cell junction genes such as TJP1 and GJA1, ultimately disrupting the intact BTB. Therefore, targeting APLN/APJ is a promising strategy to ameliorate diabetes-associated subfertility by improving the BTB integrity.

In order to reverse the harmful effects of high APLN on BTB, we tested whether a specific APJ antagonist (ML221) could alleviate the BTB disruption. Intriguingly, both in cellular and mouse in vivo models, ML221 significantly restored the expression of cell junction proteins and BTB function. Moreover, using classical diabetic *db/db* model, we showed that ML221 could efficiently improve the BTB integrity in diabetic mice, most importantly, this phenomenon was also validated in cultured human testicular tissue, indicating that manipulating APLN/APJ might be an efficient strategy to alleviate the subfertility of diabetes patients. Moreover, previous studies have shown that testes from diabetic mice cultured with ML221 can restore a certain level of testosterone expression^30^. To evaluate the ML221 effect on testosterone in our model, we performed two weeks in vivo ML221 treatment on *db/db* mice. Similarly, our results showed that a significant increase in testosterone levels in *db/db* mice after two weeks ML221 treatment (Fig. 5i), suggesting that APLN-APJ had multiple effects on regulating not only BTB but also testosterone production. Furthermore, according to immunohistochemistry results from Das et al.^30^, the expression of APLN and APJ in the testis reported seemed not cell type specific, with positive signals throughout the lumen. In our study, using immunofluorescence, the distribution of APJ was found not only in Sertoli cells, but also in other cell types as Das et al.^30^ reported. However, the expression pattern of APLN was slightly different, as our data showed that APLN was highly enriched in Sertoli cells (Fig. 2c, d) although it could be also detected throughout the seminiferous tubules. We speculated that such differences might be due to difference in mouse model, antibodies, and the experimental method.

Although we clearly indicated that targeting APJ using various methods including shRNA, ML221, or dominant negative forms of APLN significantly rescued the effect of excessive APLN, we could only partially restore the expression of cell junction genes under hyperglycemia conditions, suggesting that high glucose might induce BTB damage via other factors, albeit we proved that high glucose could directly upregulate APLN expression in TM4 cells. Furthermore, we could not determine whether Sertoli-derived or plasma-derived APLN was the major source to exacerbate BTB damage. Therefore, it is possible that targeting APLN might induce potential side effects when applied systematically^57,62,63^. Further investigation is needed to evaluate whether blocking APLN/APJ locally is a useful treatment for diabetic patients to improve their fertility.

In conclusion, our comprehensive single-cell RNA sequencing data of testes from diabetic patients uncovers an important role of APLN/APJ in regulating BTB function under hyperglycemia conditions. We, therefore, propose that targeting the APLN/APJ pathway represents a promising approach to assist male diabetic patients to improve their fertility, providing a theoretical basis of clinical application for treating diabetic-associated infertility.

## Methods

### Ethics approval

Adult human testicular samples for single-cell RNA sequencing analysis were obtained from two obstructive azoospermia males diagnosed with T2DM (DM1: 42 years old; DM2: 34 years old) undergoing sperm isolation surgery for in vitro fertilization. Adult human testicular samples for immunofluorescence and seminiferous tubule in vitro culture were obtained from three obstructive azoospermia males diagnosed with T2DM and three obstructive azoospermia males with normal spermatogenesis undergoing sperm isolation surgery for in vitro fertilization. All patients signed informed consent forms and voluntarily donated testicular tissue for this study. The experiments performed in this study were approved by Third Affiliated Hospital of Guangzhou Medical University (2017-055). The study was performed according to the guidelines of the ethics committee at the third Affiliated Hospital of Guangzhou Medical University. The study design and conduct complied with all relevant regulations regarding the use of human study participants and was conducted in accordance with the criteria set by the Declaration of Helsinki. The normal testicular single-cell data for the control group was adapted from a previously reported study, which included 9 healthy individuals^31^.

### Animals

We generated hyperglycemic mice models (HGM) according to a previous study, with slight changes^45^. In brief, 4-week-old male C57BL/6N mice purchased from Southern Medical University Laboratory Animal Center and were divided randomly into two groups, high-fat diet (HF; 60% energy as fat) and control diet. After 12 weeks of feeding, the HF group was injected intraperitoneally with 100 mg/kg body weight streptozotocin (STZ) (Sigma-Aldrich, V900890) and kept on the same diet for 6 weeks. Fasting blood glucose (6 h of fasting) was examined every week post-STZ for 4 weeks, and only glucose levels >15 mM were considered as HGM. After successful modeling, the stained pancreas and testes sections were counted. 8-week-old male *db/db* mice were purchased from Cavens Experimental Animal Co., Ltd. The procedures of animal feeding and operation were strictly carried out in accordance with the ethical guidelines (L2016149) of the Animal Ethics Committee of Southern Medical University. Mice were kept in a standard 12 h light-dark cycle in the specific-pathogen free conditions, and were permitted for free access to water and food. The ambient temperature was 20–25 °C and the humidity was 40–70%. All the mice we used were healthy and immune-normal.

### Cell culture and reagent treatment

The TM4 and HEK293T cell line (immortalized mouse Sertoli cells) was purchased from Procell Life Science Technology. TM4 and HEK293T are maintained in Dulbecco’s modified Eagle’s medium (DMEM) (GIBIO, 12100061) supplemented with 10% fetal bovine serum (VISTECH, SE100-011) and 1% penicillin and streptomycin. All cells were cultured in 37 °C humidified incubators with 5% CO2. High glucose culture environment with a glucose concentration of 9.0 g/L. The cells do not have mycoplasma contamination. All treatment times for cells were 24 h if not otherwise specified. Agonists, antagonists, and metabolites used in this study were listed as follows: Glutathione (Selleck, S4606), L-carnitine (Selleck, S2388), NAD+ (Selleck, S2518), Palmitelaidic Acid (MedChemExpress, HY-N2341), Apelin-13 (Abcam, ab141010), Pyr1-Apelin-13 (TargetMol, TP1260), Ulixertinib (Selleck, S7854), AICAR (Selleck, S1802), IDF-11774 (Selleck, S8771).

### Blood-testis barrier integrity assay

The integrity of the BTB was evaluated with a biotin tracer as described previously^46^. Briefly, 6-week-old C57BL/6N or 8-week-old *db/db* male mice were randomly divided into three groups (each group included 3 mice): Mouse in group 1 was injected with PBS as a negative control. Mouse in group 2 was injected with APLN (0.25 mg/mL, Proteintech, Ag25054). Mouse in group 3 was injected with ML221 (20 mM, Selleck, S8695). Briefly, mice from each group mentioned above were anesthetized with Sodium pentobarbital. After exposing the testes, 3 μl APLN or ML221 diluted in 50 μl PBS was injected into the testis interstitium. Besides, ML221 was injected intraperitoneally in group 3 at a dose of 10 mg per kg body weight (mg/kg), and others received the vehicle for 7 consecutive days. Then, 50 μl EZ-Link Sulfo-NHS-LC-Biotin (10 mg/mL in PBS containing 1 mM CaCl_2_, ThermoFisher, 21335) was injected for BTB integrity assay. After 30 min, the mice were humanely euthanized to collect testis.

### Blood testosterone assay

The whole blood of mice was obtained by extracting the eyeball blood under anesthesia and blood anticoagulant was added. The blood samples were centrifuged at 1200 × *g* for 5 min at 4 °C, and the plasma was collected. Concentrations of testosterone were assayed by employing commercial ELISAs (Solarbio, SEKSM-0003) in accordance with the manufacturer’s instructions.

### Sperm parameters testing

Male mice were sacrificed by cervical dislocation, and cauda epididymides were carefully dissected and squeezed on a clean slide to harvest spermatozoa, which were incubated in M2 medium (SIGMA, M7167) for sperm parameters testing by using the IVOS II (Hamilton Thorne Inc., Beverly, MA) computer-assisted sperm analysis system^64^. The concentration and motility of sperm could be automatically assessed by this system.

### Intracytoplasmic sperm injection (ICSI) and embryo transfer

ICSI was performed as described previously^65^. Cauda epididymis spermatozoa used for this experiment were collected as described previously and detached sperm heads were injected into the mature oocytes with a Piezo-driven pipette, followed by culture in KSOM medium (Millipore, MR-020P-D) in 5% CO_2_ at 37 °C. 2-cell embryos were transferred to the oviduct of CD1 pseudopregnant females. Full-term mice were delivered by cesarean section for counting live mice and implantation sites at E19.5.

### In vitro fertilization (IVF)

IVF was performed as previous described with some modifications^52^. Briefly, oocytes were collected from fallopian tubes in 200ul HFT (Easy Check, #M1130) covered with mineral oil. Spermatozoa were collected by cutting the cauda epididymis and squeezing it gently in 200 μl HFT solution covered with mineral oil, allowing the sperm to swim out freely and left for 1 h in a 34 °C, 5% CO2 incubator for capacitation. Capacitated sperm were added to the HFT drop containing oocyte pellets at a final concentration of 1 × 10^6^ sperm/ml. HFT was replaced into KSOM (Easy Check, #M1430) medium following 6 h incubation. The number of embryos at different developmental stages was counted under the microscope for statistical analysis, and in vivo transfer was performed at the blastocyst stage.

### Cell adhesion assay

The cell adhesion assay as described previously with partial modification^66^. Briefly, TM4 cells were divided into two parts, one part of the cells was completely attached to the wall and labeled with Hoechst, and a smaller number of cells were treated and labeled with cell membrane dye DiD (Beyotime Biotechnology, C1039) in red color, and finally, the ratio of red cells to blue cells was counted after washing off the suspended cells through a short three hours of cell attachment time.

### Human testicular cell isolation

After washing the testicular tissue three times with PBS, the tissue was cut up with sterile scissors and then digested with 1 mg/mL of type IV collagenase (ThermoFisher, 17104019) at 37 °C for 15 min. The digestion process was stopped with DMEM (containing 10% FBS). After centrifugation, cells were suspended in DMEM (with 10% FBS) for further sample selection.

### Human seminiferous tubule in vitro culture

After obtaining normal human testicular tissue, it was washed twice using PBS. Testicular tissue was cut into 2 mm pieces using sterile scissors and placed on 1% agarose gel. Testicular tissue was cultured using spermatogonial stem cells (SSCs) culture medium. In detail, StemPro-34 SFM (Gibco, 10640-019) with its supplement and 1% Knock out serum (Gibco, A3181502) replacement added. Supplied with 0.1 mM NEAA (Gibco, 11140076), 1 mM sodium pyruvate (Gibco, 11360070), 2 mM Glutamax (Gibco, 35050061), 1 mM Penicillin streptomycin (Gibco, 15140122), 50 μM β-mercaptoethanol (Gibco, 21985023), 1 μg/mL lactic acid (Macklin, L812422), 0.1 μg/mL vitamin (Gibco, 11120052), 10 μg/mL biotin (TargetMol, TP1116), 100 μM ascorbic acid, 60 ng/mL progesterone (Solarbio, YZ-1568007), 30 ng/mL estradiol (Solarbio, IE0220), 6 mg/mL Glucose (Gibco, 15023021), 5 mg/mL BSA (Sigma, B2064), 1% N2 supplement (ThermoFisher, 17502048), 20 ng/mL human GDNF (R&D, 212-GD-010/CF) and 10 ng/mL FGF2 (R&D, 233-FB-025), 5 mM APLN or APLN combined 10 μM ML221 solvent was added in necessary. Incubate the sample in 35 °C and change the medium every other day.

### Western-blot

TM4 cell samples were lysed and run on an SDS-PAGE gel. The primary antibodies used in this study were listed: rabbit-anti-GJA1 (Proteintech, 26980-1-AP, 1:1000), rabbit-anti-TJP1 (Proteintech, 21773-1-AP, 1:1000), rabbit-anti-APJ (Proteintech, 20341-1-AP, 1:1000), rabbit-anti-MAPK1/3 (CST, 4695T, 1:1000), rabbit-anti-p-MAPK1/3 (CST, 4370T, 1:1000), rabbit-anti-AMPKα1 (CST, 5831T, 1:1000), rabbit-anti- p-AMPKα1 (CST, 2535T, 1:1000), rabbit-anti-NCAM1 (Abcam, ab134107, 1:1000), mouse anti-SOX9 (Abcam, ab76997, 1:1000), rabbit-anti-HIF1A (Proteintech, 20960-1-AP, 1:1000), rabbit-anti-WT1 (Proteintech, 12609-1-AP, 1:1000), rabbit-anti-AR (Proteintech, 22089-1-AP, 1:1000), rabbit-anti-VIM (Proteintech, 10366-1-AP, 1:1000), and mouse anti-TUBULIN (SUNGENE, KM9007, 1:10,000). The secondary antibodies used in this study: anti-rabbit HRP (ZSJB-BIO, zb2301, 1:1000) and anti-mouse HRP (ZSJB-BIO, zb2305, 1:1000). The ECL kit (YEASON, 36208ES60) was used on the membrane before film exposure.

### ROS measurement

For TM4 cell, the medium was replaced with a new medium supplemented with Dihydroethidium (DHE, Macklin, D807594). TM4 cell were incubated with 2.5 μM DHE for 25 min in the dark at 37 °C and washed two time with PBS and then red fluorescence was detected by flow cytometry. The average of the mean fluorescence intensity for at least 3 replicates was calculated.

### Quantitative PCR (q-PCR)

Total RNA was extracted from cells using Trizol (Invitrogen, 15596026) according to the manufacturer’s instructions. cDNA was synthesized using HiScript QRT SuperMix for qPCR (Vazyme, R123-01). Quantitative PCR was performed using 2×PCR Master Mix (GenStar, A301-10) and expression levels of target genes were normalized to the expression of the *Actb* and were calculated based on the comparative cycle threshold method (2^−△△Ct^). Error bars are mean ± SD from three independent experiments. The primer sequences used in this study were listed as follows: ms-*Tjp1*, F: GAGCGGGCTACCTTACTGAAC, R: GTCATCTCTTTCCGAGGCATTAG, ms-*Gja1*, F: ACAGCGGTTGAGTCAGCTTG, R: GAGAGATGGGGAAGGACTTGT, ms-*Ncam1*, F: ACCACCGTCACCACTAACTCT, R: TGGGGCAATACTGGAGGTCA, ms-*Cldn11*, F: ATGGTAGCCACTTGCCTTCAG, R: AGTTCGTCCATTTTTCGGCAG, ms-*Actb*, F: GGCTGTATTCCCCTCCATCG, R: CCAGTTGGTAACAATGCCATGT.

### shRNA transfection

Plasmids encoding shRNAs were obtained from the Tsingke Biotechnology. HEK293T cells were transfected with APJ-shRNA#1, APJ-shRNA#3, NC-shRNA, and packaging plasmids (psPAX2 and pMD2.G). The cells do not have mycoplasma contamination. Media containing lentiviruses were collected 48 h after transfection. Harvested media were added to TM4 cells for further experiments. The following shRNAs were used in this study: mouse *Apj* shRNA-1: 5′- ATAAATGAGGGAAGGTTATAT-3′, mouse *Apj* shRNA-4: 5′- CGATTCCCTATAGTCAAGAAA-3′, mouse NC-shRNA: 5′- GGTTCTCCGAACGTGTCACGT-3′.

### Histological examination

Testicular tissue from normal fertility and type 2 diabetes mellitus (T2DM) donors was fixed in 4% paraformaldehyde for 12 h on a room temperature shaker and routinely paraffin-embedded before sectioning. Prior to staining, tissue sections were dewaxed in xylene and rehydrated by decreasing concentrations of ethanol. The sections were then stained with hematoxylin (Mayer’s hematoxylin solution) and eosin (H&E). After staining, the sections were dehydrated using ethanol and xylene and finally fixed using resin. Sections were observed under an OLYMPUS BX51 microscope with a 40× oil microscope.

### Immunofluorescence

Testis tissues were fixed with 4% paraformaldehyde 6 h at room temperature, then embedded in paraffin and cut into 5μm sections for immunostaining after deparaffinization and rehydration. Testicular sections were washed three times with PBST (0.3% TritonX-100 in PBS) and blocked with 3% BSA at room temperature for 1 h. Paraffin sections of human testes were first dewaxed and subjected to antigen retrieval, followed by blocking with 3% BSA for 1 h. Primary antibodies were incubated overnight at 4 °C. Subsequently, they were washed three times with PBS and then incubated with secondary antibody or/and peanut agglutinin (PNA) for 1 h at room temperature. Nuclei were counterstained with 10 mg/mL of Hoechst 33342 for 15 min at room temperature and then washed with PBS. Images were taken with a ZEISS LSM880 confocal microscope. The primary antibodies used in this study were listed: rabbit-anti-GJA1 (Abcam, ab217676, 1:200), rabbit-anti-TJP1 (Abcam, ab221547, 1:400), rabbit-anti-CDH2 (Abcam, ab18203, 1:100), rabbit-anti-NCAM1 (Abcam, ab220360, 1:100), rabbit-anti-HIF1A (Proteintech, 20960-1-AP, 1:50), mouse-anti-ACTB (Proteintech, 66009-1-Ig, 1:400), mouse-anti-VIMENTIN (Proteintech, 60330-1-Ig, 1:50), mouse anti-DDX4 (Abcam, ab27591, 1:500), rabbit anti-SOX9 (Millipore, AB5535, 1:200), rabbit anti-SYCP3 (Abcam, ab15093, 1:400), mouse anti-CREM (Santa, sc-390426, 1:50), rabbit anti-KIT (Abcam, ab32363, 1:200), mouse anti-FGFR3 (Santa, sc-13121, 1:30), rabbit anti-STRA8 (Millipore, ABN1656, 1:100), mouse anti-γH2AX (Abcam, ab185619, 1:400), rabbit anti-INSL3 (Novus Biologicals, NBP1-81223, 1:300), mouse anti-MKI67 (Ascend Biotechnology, AM0146, 1:200).

### Quantification of immunofluorescence

Immunofluorescent images of cell or testis were quantified using ImageJ^67^. The quantification of immunofluorescence with ImageJ as described previously with partial modification^68^. Briefly, images were converted to 8 bits and checked the area, mean gray value and min&max gray value in the set measurement options. Used the freehand tool to tick the area of positive cells and calculate the relative fluorescence intensity. For immunofluorescence images of paraffin sections, sections from different patients are randomly selected from at least five fields of view for positive cell signal intensity quantification. For cell samples, positive signal intensities on at least 100 cells will be counted.

### single-cell RNA sequencing library preparation and sequencing

After sample collection, a mouth pipette was used to immediately transfer the single cell into prepared lysis buffer with an 8-nt barcode. The preparation of single-cell RNA sequencing library was performed using the STRT-seq method^69^. In brief, samples were incubated at 72 °C for 3 min after vortexing for 60 s. Then, the first-strand cDNA was reverse-synthesized using oligo (dT) anchored with cell-specific barcode, unique molecular identifiers (UMIs) and template-switching oligonucleotides (TSO). After that, the second-strand cDNAs were synthesized, and the cDNAs were amplified by 18 cycles of PCR. Subsequently, the products of single cells were pooled and purified. Next, the PCR product was further amplified using biotinylated pre-indexed primers, and four more cycles of PCR were performed to introduce a biotin tag at the 3′ end of the amplified cDNA. About 300 ng of cDNA was then sheared with a Covaris M220 to obtain fragments with an average length of approximately 300 bp. Dynabeads MyOne Streptavidin C1 beads (Thermo Fisher, 65002) were used to enrich for the 3′ terminal cDNAs. The RNA-seq library was then constructed using a Kapa Hyper Prep Kit (Kapa Biosystems, KK8504) and subjected to 150 bp paired-end sequencing on an Illumina HiSeq XTEN platform (sequenced by Novogene).

### CUT&Tag library preparation

In order to study the distribution of HIF1A in TM4 cells, we used NovoNGS ® CUT&Tag 2.0 High-Sensitivity Kit (Novoprotein, N259-YH01) to capture HIF1A-binding sites. The experimental process was performed according to the manufacturer’s instructions. In brief, 1 × 10^5^ TM4 cells were prepared and immobilized on concanavalin A beads. Beads are incubated with a HIF1A primary antibody (Proteintech, 20960-1-AP), followed by incubation with a secondary antibody anti-Rabbit IgG (Abcam, ab6702). The concanavalin A beads immobilized with cells were washed twice and pA-Tn5 was added. Tn5 was activated by addition of Mg^2+^ and incubated at 37 °C for 1 h. Reactions were stopped by the addition of 10 µL 0.5 M EDTA, 3 µL 10% SDS and 2.5 µL 20 mg/mL Proteinase K to each sample. Phenol-chloroform was used to extract DNA and constructed CUT&Tag library according to the manufacturer’s instructions. Library was quantified by Equalbit dsDNA HS Assay Kit (Vazyme, EQ111-01) using Qubit™ 4 Fluorometer (Invitrogen, Q33226). Libraries were subjected to paired-end 150 bp sequencing on NovaSeq platform at Novogene.

### Single-cell RNA sequencing data processing

Single-cell RNA sequencing raw reads were processed to remove Template Switch Oligo (TSO) sequence and polyA tail sequence. And then the reads with adapter and low-quality bases were removed to obtain clean reads. The clean reads were aligned to human genome (GRCh38, gencode version) using Hisat2 (v 2.1.0)^70^ with default parameters, and uniquely mapped reads according to unique molecular identifiers (UMI) numbers were counted using HTSeq (v 0.11.3)^71^. For all 960 sequenced single cells, we quantified the numbers of genes and transcripts in each cell. Cells with either fewer than 2000 genes or fewer than 10,000 UMI detected were removed from downstream analysis. After filtering, we obtained 441 cells from DM donor 1 and 459 cells from DM donor 2. The expression levels were normalized by log_2_(TPM/10+1) as described previously^31^. Once the TPM was higher than 0, we consider that this gene is detected in the individual cell.

### Data integration analysis

In order to more accurately define the cell type of diabetic spermatogenesis data, we used the integrated analysis strategy in Seurat3^32^ to integrate our previously published normal spermatogenesis data^31^ with diabetic spermatogenesis data. To avoid the batch effect caused by the different analysis process, we processed the raw data of normal spermatogenesis and kept the same as the data of diabetes. FindIntegrationAnchors and IntegrateData function in Seurat3 were used to integrate different datasets. PCs 1–19 were selected to perform integrate and UMAP analysis. We used FindConservedMarkers function to find conservative markers to identify cell type on the integrated dataset.

### Identification of DEGs

To identify DEGs between normal and diabetic patients in each specific cell type, we use the Seurat FindMarker function based on normalized TPM expression values. A Wilcox-test was used to calculate *p* values and fold-change. Only genes with an average log-transformed difference greater than 0.5, a *p* value less than 0.05 were defined as DEGs. It should be noted that the DEG analysis was performed separately for the two DM patients. If a gene meets the DEG threshold condition in both patients, it will be retained for the next step of analysis. The metabolism-relevant gene was obtained from the KEGG database. GO analysis was performed with Metascape (www.metascape.org) using default parameters.

### GSEA analysis

We used Gene Set Enrichment Analysis (GSEA) to identify gene sets that showed significant differences between normal and diabetic patients in each specific cell type. gseKEGG function in clusterProfiler R package^72^ was used to perform GSEA analysis. We selected the default setting, a threshold *p* value < 0.05.

### Pseudotime analysis

Pseudotime was generated by Monocle3^33^ based on 2,000 highly variable genes identified by Seurat. The coordinates of UMAP in Monocle3 were also based on the results of Seurat integrate analysis.

### Metabolic pathway activities analysis

We used “Single-Cell-Metabolic-Landscape” algorithm^34^ to calculate metabolic pathway activity. The pipeline and code used in this analysis are available at GitHub page of the Locasale Lab: https://github.com/LocasaleLab/Single-Cell-Metabolic-Landscape.

### CUT&Tag data analysis

The processing of CUT&Tag data was similar to what we described before^73^. In brief, the adaptor sequence were removed using bbduk (v 38.18) and low-quality reads were trimmed by trimmomatic^74^ (v 0.39), with the length cut-off 35 bp. The clean reads were aligned to GRCm38 genome (Ensembl version) using bowtie2 (v 2.3.5.1)^75^ with the options (--very-sensitive --end-to-end). Then, low quality mapping reads were removed using samtools with the option (-q 35). MACS2 (v 2.2.6)^76^ was used to call narrow peaks with the options (-g mm -f BAMPE -B --call-summits). Bigwig files were generated using genomeCoverageBed from bedtools (v 2.29.2) (scale factor = 20 million/<each sample’s total unique read>) and bedGraphToBigWig (v 2.8).

### Ligand-receptor-target links analysis

The ligand-receptor-target links analysis was completed by two R packages, CellCall^48^ and NicheNet^49^. Briefly, CellCall package was based on the ligand-receptor-transcript factor (L-R-TF) axis datasets in KEGG, and infers intercellular communication by combining the expression of ligands/receptors and downstream TF activities for certain L-R pairs. NicheNet package used gene expression data between interacting cells and known signal transduction networks to construct the connection relationship between ligand and target genes, and can predict which ligands produced by one cell type regulate the expression of which target genes in another cell type.

For CellCall analysis, in order to identify the strongest interaction between Sertoli cells and which kind of germ cells, we input the expression matrix (TPM) of all germ cells and Sertoli cells in the normal spermatogenesis data to CellCall package. TransCommuProfile and ViewInterCircos functions with default setting were used to infer cell-cell communication score and generate circle plot. Finally, we found that Sertoli cells have the strongest ability to interact with spermatogonia cells.

For NicheNet analysis, we merge the four types of cells belonging to the spermatogonial stage, SSC_1, SSC_2, SPG_ing, and SPG_ed, into SPG group. Nichenet_seuratobj_aggregate function was used to connect the results of Seurat with the analysis of NicheNet. We defined Sertoli cells as “sender/niche” cell population, and SPG cells as “receiver/target” cell population to perform NicheNet analysis. Genes with fold change ≥ 2 were selected into the NicheNet as a gene set of interest which were potentially affected by ligands expressed by interacting cells.

### Untargeted metabolomic

After digestion of treated TM4 cells using trypsin, the cells were washed twice in PBS and immediately snap-frozen in liquid nitrogen. Four biological replicate samples were collected for both the experimental and control groups. The processed cells were taken for further analysis on LC–MS/MS platform (Bioprofile Co. Ltd., Shanghai, China).

### Statistics and reproducibility

All the H&E, immunofluorescence, ICSI, IVF and Western-blot were conducted in at least three biological replicates to ensure the reproducibility. GraphPad Prism 9 was used to analyze the data of qPCR, immunostaining and FACS. S. Box & whisker plot limits indicate the minimum and maximum, and boxplot center line indicates the median. Statistical significance is presented in the figures as *p < 0.05, **p < 0.01, ***p < 0.001, ****p < 0.0001, and not significant (ns). For comparisons between 2 groups, such as qPCR, unpaired two-tailed student’s t test was used. For comparisons among multiple groups, one-way ANOVA was used.

### Reporting summary

Further information on research design is available in the Nature Portfolio Reporting Summary linked to this article.

## Supplementary information

Supplementary Information

Description of Additional Supplementary Files

Supplementary Data 1

Supplementary Data 2

Reporting Summary

## Data Availability

Raw sequencing data for human normal testis was retrieved from Gene Expression Omnibus (GEO) under accession number GSE106487. The single-cell RNA sequencing raw data from human diabetic patients’ testis in this paper have been deposited in the Genome Sequence Archive in National Genomics Data Center, China National Center for Bioinformation / Beijing Institute of Genomics, Chinese Academy of Sciences (GSA-Human: HRA000976) that are publicly accessible at https://ngdc.cncb.ac.cn/gsa-human/browse/HRA000976. The HIF1A CUT&Tag raw data in this paper have been deposited in the Genome Sequence Archive (GSA: CRA004696) that are publicly accessible at https://ngdc.cncb.ac.cn/gsa/browse/CRA004696. The processed data in this paper have been deposited in the Gene Expression Omnibus (GEO) at NCBI under accession number GSE179080. Source data are provided with this paper.

## Source data

Source Data

## Code availability

The code used in this paper is deposited at https://github.com/YangXinyan/DM-scRNA-seq (10.5281/zenodo.7261195).
