# Supplementary material for: Targeting APLN/APJ restores blood-testis barrier and improves spermatogenesis in murine and human diabetic models
Source: Nat Commun. 2022 Nov 28;13:7335. doi: 10.1038/s41467-022-34990-3 (PMC9705293; doi:10.1038/s41467-022-34990-3)
Supplement: Supplementary file 1 — Supplementary Information [file 41467_2022_34990_MOESM1_ESM.pdf]

Supplementary Information

Targeting APLN/APJ restores blood-testis barrier and improves spermatogenesis in murine and human diabetic models

Ke Song, Xinyan Yang, Geng An, Xinyu Xia, Jiexiang Zhao, Xiaoheng Xu, Cong Wan, Tianyuan Liu, Yi Zheng, Shaofang Ren, Mei Wang, Gang Chang, Shane J.F. Cronin, Josef M. Penninger, Tao Jing, Xianghong Ou, Shuan Rao, Zhaoting Liu, Xiao-Yang Zhao

*Corresponding authors: Shuan Rao (raoshuan1@smu.edu.cn), Zhaoting Liu (liuzhaoting@i.smu.edu.cn), Xiao-Yang Zhao (zhaoxiaoyang@smu.edu.cn).

Contents for Supporting Information

I. Supplementary Figures

a

Batch	Type	Age of donor	Strategy	Cell Number (After QC: 900)
DM1	T2DM	42	Random	441
DM2	T2DM	34	Random	459

b

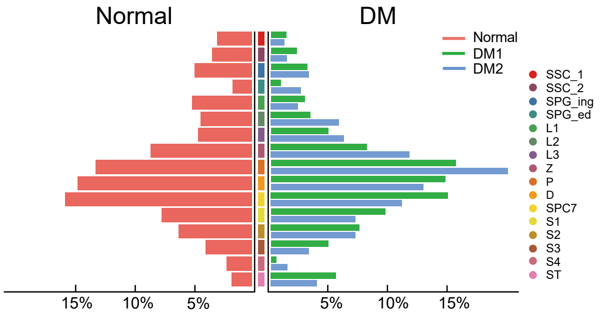

c

	SSC_1	SSC_2	SPG_ing	SPG_ed	L1	L2	L3	Z	P	D	SPC7	S1	S2	S3	S4	ST	LD	T
Normal	74	85	122	42	128	109	114	216	332	370	396	192	156	99	54	43	91	187
DM1	5	6	14	11	10	25	27	51	87	56	47	31	31	14	6	17	3	NA
DM2	6	10	14	4	13	19	22	37	71	67	68	44	34	22	2	25	1	NA

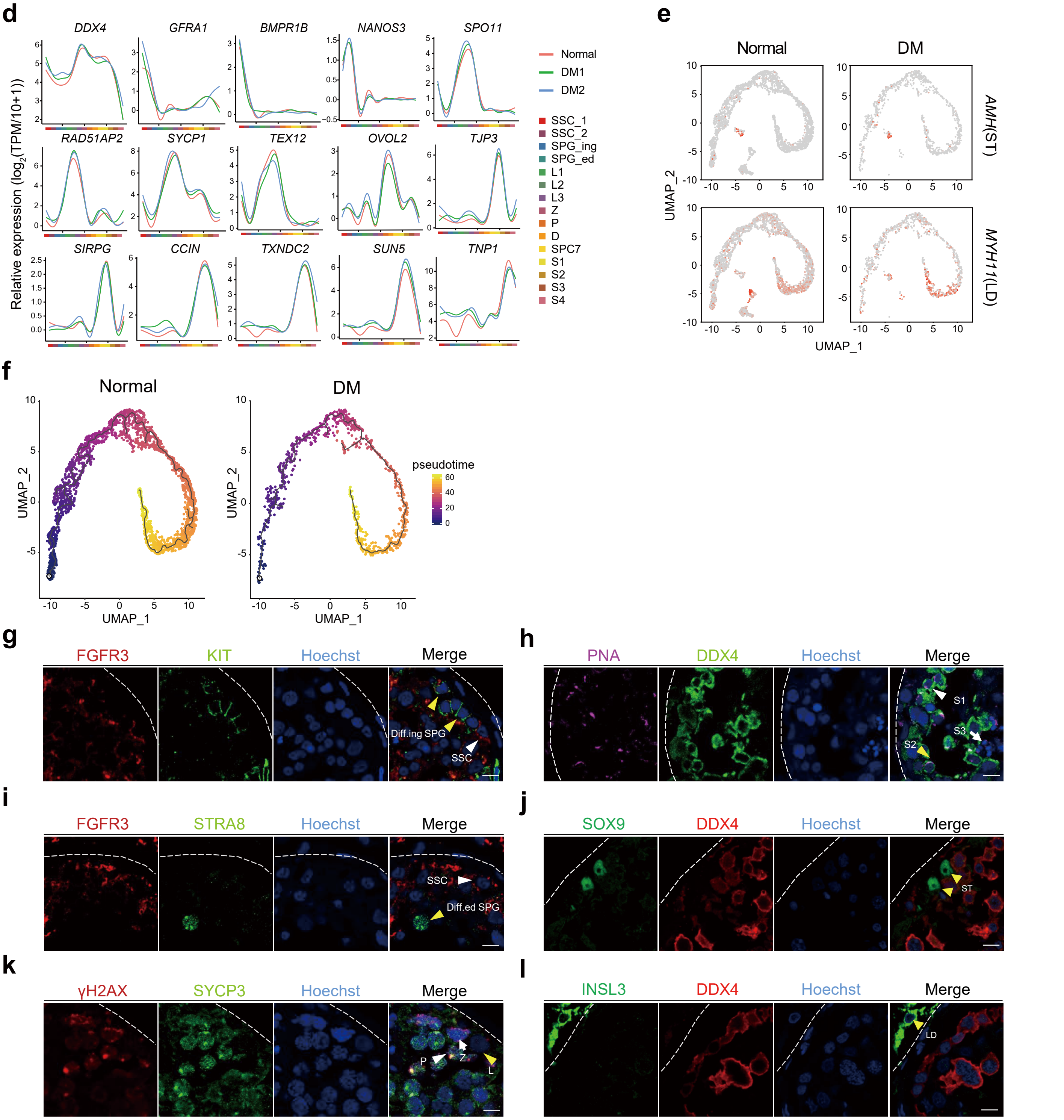

Supplementary Figures. 1| Comparison of healthy and diabetic testis at single cell resolution. a, Sample information of diabetic patients' testicular cells analyzed in this study. **b**, Bar graph showing the ratio of each cluster in spermatogenesis between normal and diabetic patients. **c**, Cell number of each cluster in spermatogenesis between normal and diabetic patients. **d**, Line plots showing the similar relative expression patterns of the potential marker gene of each cluster between normal and diabetic patients. **e**, Gene expression patterns of somatic marker genes on UMAP plots. A gradient of gray, red indicates low to high expression levels. **f**, Developmental pseudotime of adult human male germ cells from donors with normal fertility (left) and diabetic patients (right). Black line indicates the developmental path of these cells. **g**, Immunofluorescence of FGFR3 (red) co-stained with KIT (green) in diabetic patients' testicular paraffin sections. Scale bar, 10 μm. Yellow arrowheads indicated Diff.ing SPG, white arrowhead indicates SSC. **h**, Immunofluorescence of PNA (pink) co-stained with DDX4 (green) in diabetic patients' testicular paraffin sections. Scale bar, 10 μm. White arrowhead S1, yellow arrowhead indicated S2, and arrows indicated S3. **i**, Immunofluorescence of FGFR3 (red) co-stained with STRA8 (green) in diabetic patients' testicular paraffin sections. Yellow arrowhead indicates Diff.ed SPG, White arrowhead indicates SSC. Scale bar, 10 μm. **j**, Immunofluorescence of DDX4 (red) co-stained with SOX9 (green) in diabetic patients' testicular paraffin sections. Yellow arrowhead indicates Sertoli cells (ST). Scale bar, 10 μm. **k**, Immunofluorescence of γH2AX (red) co-stained with SYCP3 (green). Yellow arrowhead indicates leptotene (L) spermatocytes, white arrowhead indicates zygotene (Z) spermatocytes, and white arrowhead indicates pachytene (P). Scale bar, 10 μm. **l**, Immunofluorescence of DDX4 (red) co-stained with INSL3 (green) in diabetic patients' testicular paraffin sections. Scale bar, 10 μm. Yellow arrowhead indicates Leydig cells (LD).

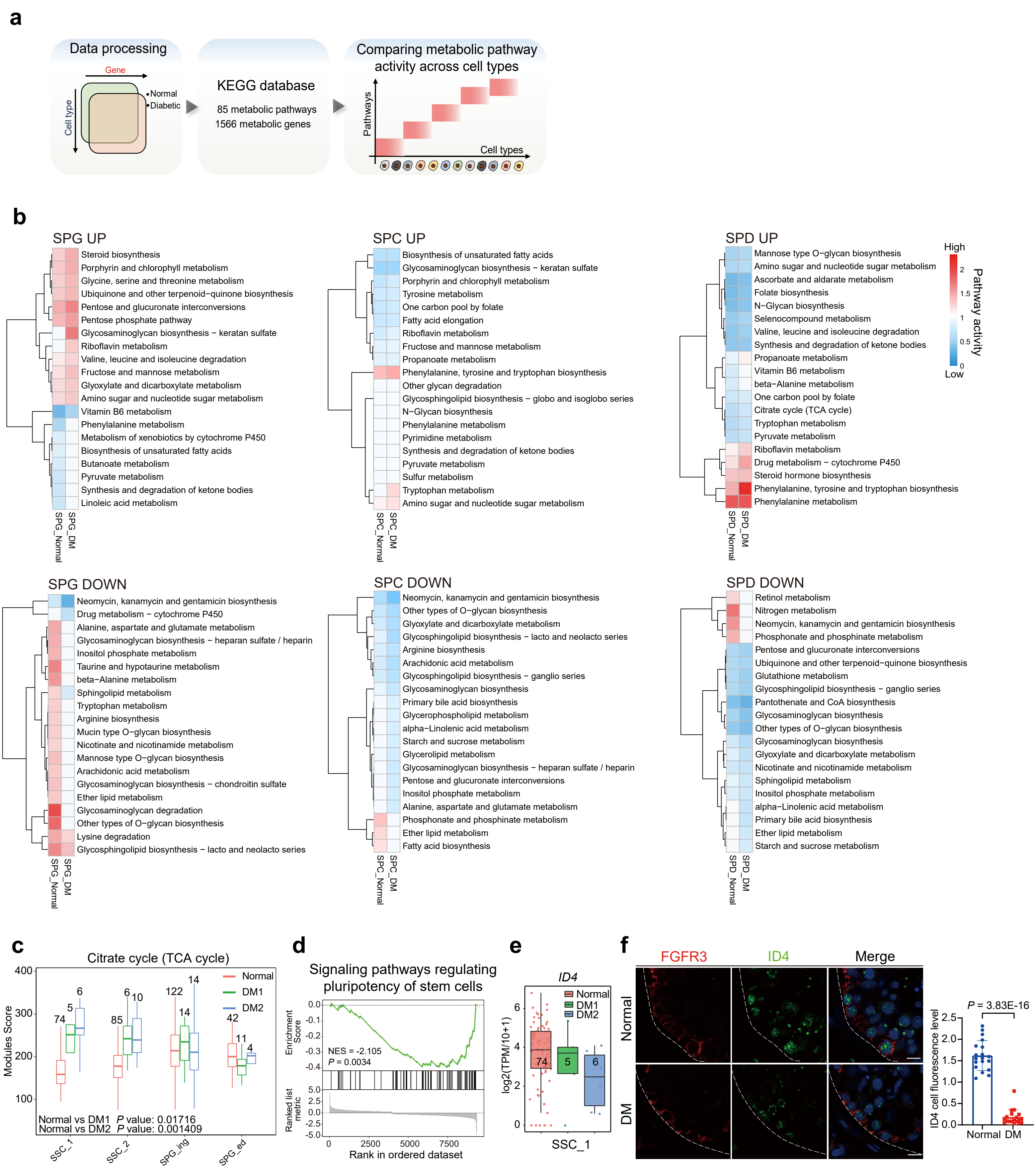
a
Data processing
Gene
Cell type
Normal
Diabetic
KEGG database
85 metabolic pathways
1566 metabolic genes
Comparing metabolic pathway activity across cell types
Pathways
Cell types
b
SPG UP
Steroid biosynthesis
Porphyrin and chlorophyll metabolism
Glycine, serine and threonine metabolism
Ubiquinone and other terpenoid−quinone biosynthesis
Pentose and glucuronate interconversions
Pentose phosphate pathway
Glycosaminoglycan biosynthesis − keratan sulfate
Riboflavin metabolism
Valine, leucine and isoleucine degradation
Fructose and mannose metabolism
Glyoxylate and dicarboxylate metabolism
Amino sugar and nucleotide sugar metabolism
Vitamin B6 metabolism
Phenylalanine metabolism
Metabolism of xenobiotics by cytochrome P450
Biosynthesis of unsaturated fatty acids
Butanoate metabolism
Pyruvate metabolism
Synthesis and degradation of ketone bodies
Linoleic acid metabolism
SPG_Normal
SPG_DM
SPC UP
Biosynthesis of unsaturated fatty acids
Glycosaminoglycan biosynthesis − keratan sulfate
Porphyrin and chlorophyll metabolism
Tyrosine metabolism
One carbon pool by folate
Fatty acid elongation
Riboflavin metabolism
Fructose and mannose metabolism
Propanoate metabolism
Phenylalanine, tyrosine and tryptophan biosynthesis
Other glycan degradation
Glycosphingolipid biosynthesis − globo and isoglobo series
N−Glycan biosynthesis
Phenylalanine metabolism
Pyrimidine metabolism
Synthesis and degradation of ketone bodies
Pyruvate metabolism
Sulfur metabolism
Tryptophan metabolism
Amino sugar and nucleotide sugar metabolism
SPC_Normal
SPC_DM
SPD UP
Mannose type O−glycan biosynthesis
Amino sugar and nucleotide sugar metabolism
Ascorbate and aldarate metabolism
Folate biosynthesis
N−Glycan biosynthesis
Selenocompound metabolism
Valine, leucine and isoleucine degradation
Synthesis and degradation of ketone bodies
Propanoate metabolism
Vitamin B6 metabolism
beta−Alanine metabolism
One carbon pool by folate
Citrate cycle (TCA cycle)
Tryptophan metabolism
Pyruvate metabolism
Riboflavin metabolism
Drug metabolism − cytochrome P450
Steroid hormone biosynthesis
Phenylalanine, tyrosine and tryptophan biosynthesis
Phenylalanine metabolism
SPD_Normal
SPD_DM
High
Low
Pathway activity
2
1.5
1
0.5
0
SPG DOWN
Neomycin, kanamycin and gentamicin biosynthesis
Drug metabolism − cytochrome P450
Alanine, aspartate and glutamate metabolism
Glycosaminoglycan biosynthesis − heparan sulfate / heparin
Inositol phosphate metabolism
Taurine and hypotaurine metabolism
beta−Alanine metabolism
Sphingolipid metabolism
Tryptophan metabolism
Arginine biosynthesis
Mucin type O−glycan biosynthesis
Nicotinate and nicotinamide metabolism
Mannose type O−glycan biosynthesis
Arachidonic acid metabolism
Glycosaminoglycan biosynthesis − chondroitin sulfate
Ether lipid metabolism
Glycosaminoglycan degradation
Other types of O−glycan biosynthesis
Lysine degradation
Glycosphingolipid biosynthesis − lacto and neolacto series
SPG_Normal
SPG_DM
SPC DOWN
Neomycin, kanamycin and gentamicin biosynthesis
Other types of O−glycan biosynthesis
Glyoxylate and dicarboxylate metabolism
Glycosphingolipid biosynthesis − lacto and neolacto series
Arginine biosynthesis
Arachidonic acid metabolism
Glycosphingolipid biosynthesis − ganglio series
Glycosaminoglycan biosynthesis
Primary bile acid biosynthesis
Glycerophospholipid metabolism
alpha−Linolenic acid metabolism
Starch and sucrose metabolism
Glycerolipid metabolism
Glycosaminoglycan biosynthesis − heparan sulfate / heparin
Pentose and glucuronate interconversions
Inositol phosphate metabolism
Alanine, aspartate and glutamate metabolism
Phosphonate and phosphinate metabolism
Ether lipid metabolism
Fatty acid biosynthesis
SPC_Normal
SPC_DM
SPD DOWN
Retinol metabolism
Nitrogen metabolism
Neomycin, kanamycin and gentamicin biosynthesis
Phosphonate and phosphinate metabolism
Pentose and glucuronate interconversions
Ubiquinone and other terpenoid−quinone biosynthesis
Glutathione metabolism
Glycosphingolipid biosynthesis − ganglio series
Pantothenate and CoA biosynthesis
Glycosaminoglycan biosynthesis
Other types of O−glycan biosynthesis
Glycosaminoglycan biosynthesis
Glyoxylate and dicarboxylate metabolism
Nicotinate and nicotinamide metabolism
Sphingolipid metabolism
Inositol phosphate metabolism
alpha−Linolenic acid metabolism
Primary bile acid biosynthesis
Ether lipid metabolism
Starch and sucrose metabolism
SPD_Normal
SPD_DM
c
Citrate cycle (TCA cycle)
Modules Score
400
300
200
100
Normal
DM1
DM2
74
5
6
85
6
10
122
14
14
42
11
4
Normal vs DM1 P value: 0.01716
Normal vs DM2 P value: 0.001409
SSC_1
SSC_2
SPG_ing
SPG_ed
d
Signaling pathways regulating pluripotency of stem cells
Enrichment Score
0.0
-0.1
-0.2
-0.3
-0.4
NES = -2.105
P = 0.0034
Ranked list metric
5.0
2.5
0.0
-2.5
-5.0
0
2500
5000
7500
Rank in ordered dataset
e
ID4
log2(TPM/10+1)
6
4
2
0
Normal
DM1
DM2
74
5
6
SSC_1
f
FGFR3
ID4
Merge
Normal
DM
P = 3.83E-16
ID4 cell fluorescence level
2.5
2.0
1.5
1.0
0.5
0.0
Normal
DM

Supplementary Figures. 2| Metabolic difference in SPG, SPC and SPD stages and metabolic disorders associated with diabetes affect the pluripotency of spermatogonia. **a**, Schematic illustration of the metabolism related genes analysis workflow. **b**, Metabolic pathway activities in the three major germ cell types (SPG, SPC, SPD) from the normal and diabetic patients single-cell RNA sequencing data. **c**, Boxplot showing the differences in the expression of Citrate cycle (TCA cycle) between normal and diabetic patients at different spermatogonia stages. The y axis represents the sum of the TPM of all genes in this gene set. The cell numbers at each stage are indicated above each box. Each box represents the median and the 25% and 75% quartiles, and the whiskers indicate 1.5 times of the interquartile range. *P* value was calculated by wilcoxon rank sum test. **d**, GSEA analysis showing the down-regulated pathway activity in spermatogonia cells of diabetic patients. *P* value was calculated by permutation test. **e**, Boxplot showing the expression levels of *ID4* in SSC_1 stage. The cell numbers at each stage are indicated above each box. Each box represents the median and the 25% and 75% quartiles, and the whiskers indicate 1.5 times of the interquartile range. The cell numbers at each stage are indicated above each box. **f**, Immunofluorescence of FGFR3 (red) co-stained with ID4 (green) in normal and diabetic patients' testicular paraffin sections. n = 3 per group. Mean ± SEM. Representative immunofluorescence images and quantitative analysis of ID4. Two-tailed student's t test was performed. Scale bar, 10 μm.

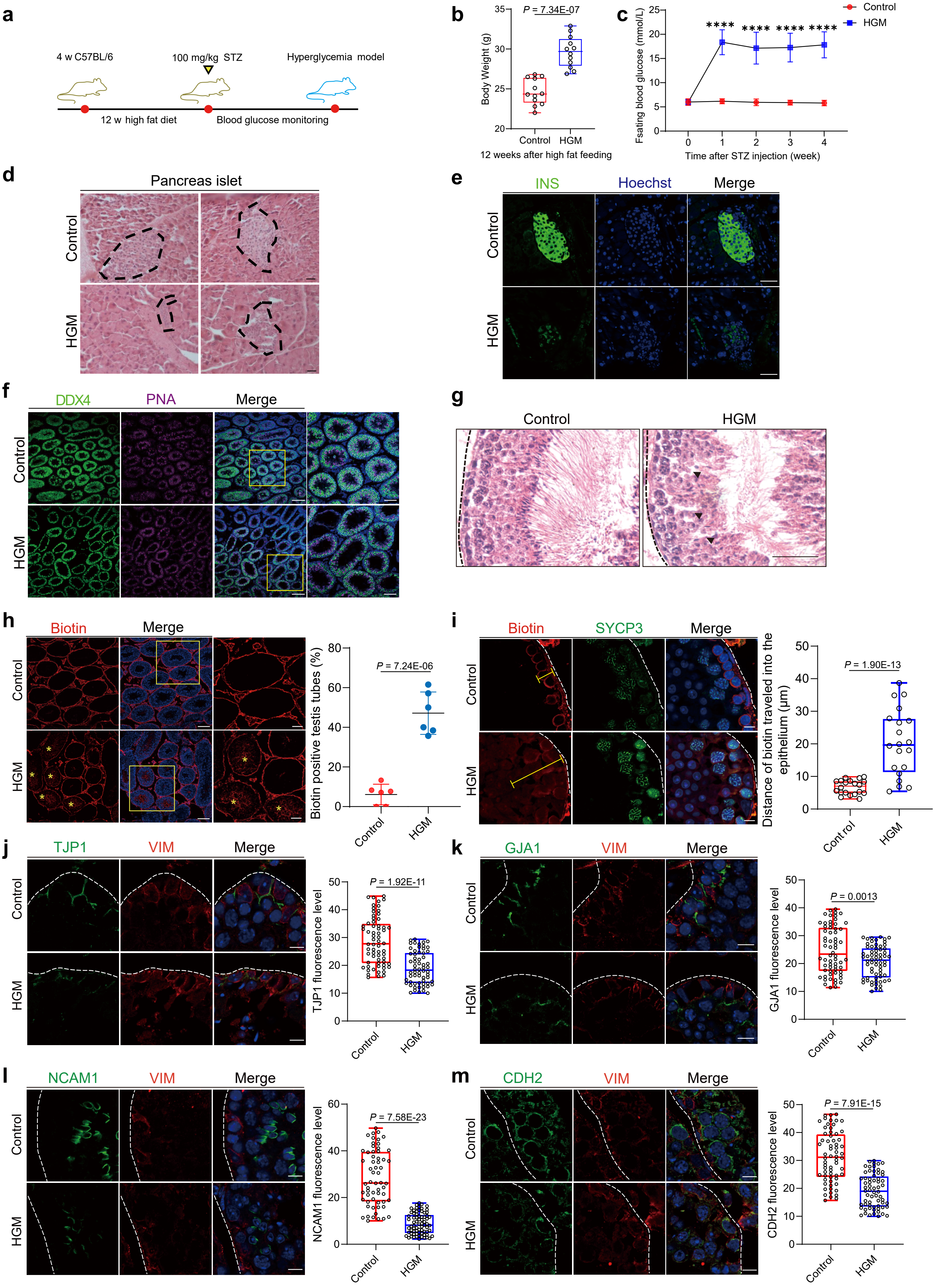
a
4 w C57BL/6
100 mg/kg STZ
Hyperglycemia model
12 w high fat diet
Blood glucose monitoring
b
P = 7.34E-07
Body Weight (g)
Control
HGM
12 weeks after high fat feeding
c
Control
HGM
****
Fsating blood glucose (mmol/L)
Time after STZ injection (week)
d
Pancreas islet
Control
HGM
e
INS
Hoechst
Merge
f
DDX4
PNA
Merge
g
Control
HGM
h
Biotin
Merge
P = 7.24E-06
Biotin positive testis tubes (%)
i
Biotin
SYCP3
Merge
P = 1.90E-13
Distance of biotin traveled into the epithelium (μm)
j
TJP1
VIM
Merge
P = 1.92E-11
TJP1 fluorescence level
k
GJA1
VIM
Merge
P = 0.0013
GJA1 fluorescence level
l
NCAM1
VIM
Merge
P = 7.58E-23
NCAM1 fluorescence level
m
CDH2
VIM
Merge
P = 7.91E-15
CDH2 fluorescence level

Supplementary Figures. 3| Loss of BTB integrity in induced hyperglycemia murine model (HGM). a, Schematic illustration of the hyperglycemia mouse model. **b**, Body weight between control and HGM after 12 weeks high fat feeding. Box-and-whisker plots denote the maximum (top whisker), 75th (top edge of box), 25th (bottom edge of box) and minimum (bottom whisker) percentiles, and the median (line in box). n = 12 mice per group. **c**, Fasting blood glucose between control and HGM after STZ injection. Mean ± SEM. ****p< 0.0001. n = 12 mice per group. **d**, H&E staining of pancreatic sections in control and HGM. The area marked by the dotted line is the islet. Scale bar, 20 μm. **e**, Immunofluorescence of INSULIN (green) in control and HGM pancreatic sections. Scale bar, 50 μm. **f**, Immunofluorescence of PNA (pink) co-stained with DDX4 (green) in control and HGM testicular paraffin sections. Scale bar, 100 μm. The yellow rectangular box shows a partial magnification of the testis. **g**, H&E staining of testicular paraffin sections in control and HGM. Scale bar, 50 μm. Black arrows show obvious vacuoles in tubule. **h**, Immunofluorescence of biotin (red) between control and HGM testicular paraffin sections. Scale bar, 50 μm. Biotin positive seminiferous tubules percentage in control and HGM. Mean ± SEM. Unpaired two-tailed t test. Statistics were performed in six mouse testes each group (n=6). **i**, Immunofluorescence of biotin (red) co-stained with SYCP3 (green) in control and HGM testicular paraffin sections. Distance of biotin penetrated into the epithelium were counted in control and HGM. Scale bar, 10 μm. Box-and-whisker plots denote the maximum (top whisker), 75th (top edge of box), 25th (bottom edge of box) and minimum (bottom whisker) percentiles, and the median (line in box). Data are presented as means ± SEM. Unpaired two-tailed t test. **j-m**, Immunofluorescence of VIM (red) co-stained with TJP1, GJA1, NCAM1 and CDH2 (green) in control and HGM testicular paraffin sections. n = 3 per group. Box-and-whisker plots denote the maximum (top whisker), 75th (top edge of box), 25th (bottom edge of box) and minimum (bottom whisker) percentiles, and the median (line in box). Representative immunofluorescence images and quantitative analysis of TJP1, GJA1, NCAM1 and CDH2. Scale bar, 10 μm. Two-tailed student's t test was performed.

a **b**

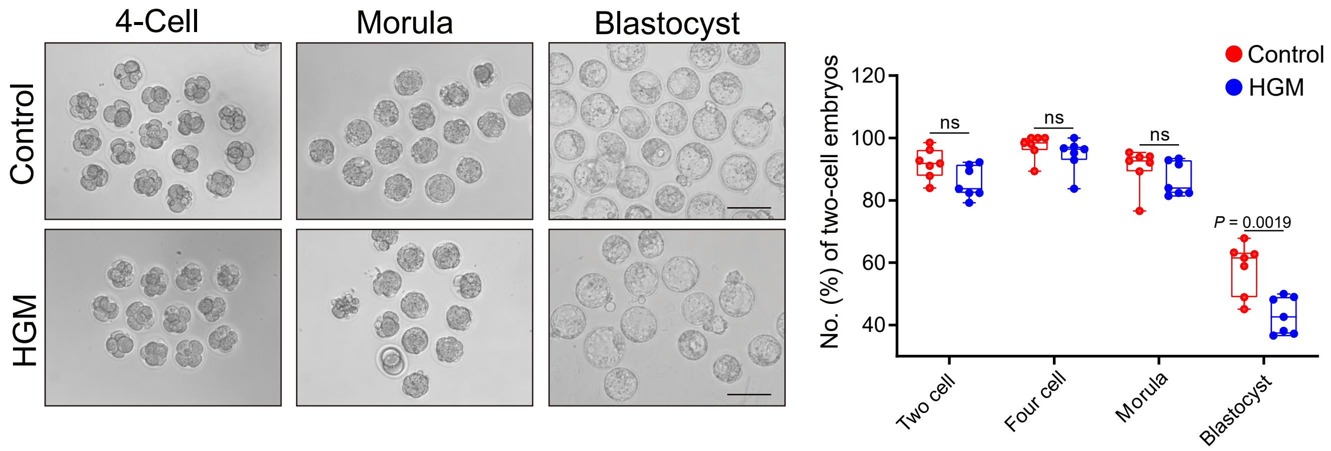

c

Origin of sperm	No. of Injected oocytes	No. (%) of two-cell embryos	No. (%) of two-cell embryos cultured in vitro	No. (%) of blastocysts
Control	350	324 (92.0%)	324	171 (52.7%)
HGM	355	300 (84.5%)	300	99 (33.0%)

Supplementary Figures. 4| Decreased sperm quality in hyperglycemia murine model.

a, Brightfield diagram of 4-Cell, Morula and Blastocyst between control and HGM. Scale bar, 1 mm. **b**, Two cell, Four cell, Morula and Blastocyst development rate in control and HGM. Unpaired two-tailed t test. n = 6 independent experiments. Box-and-whisker plots denote the maximum (top whisker), 75th (top edge of box), 25th (bottom edge of box) and minimum (bottom whisker) percentiles, and the median (line in box). **c**, The trilinear table shows all the embryo injection data in control and HGM.

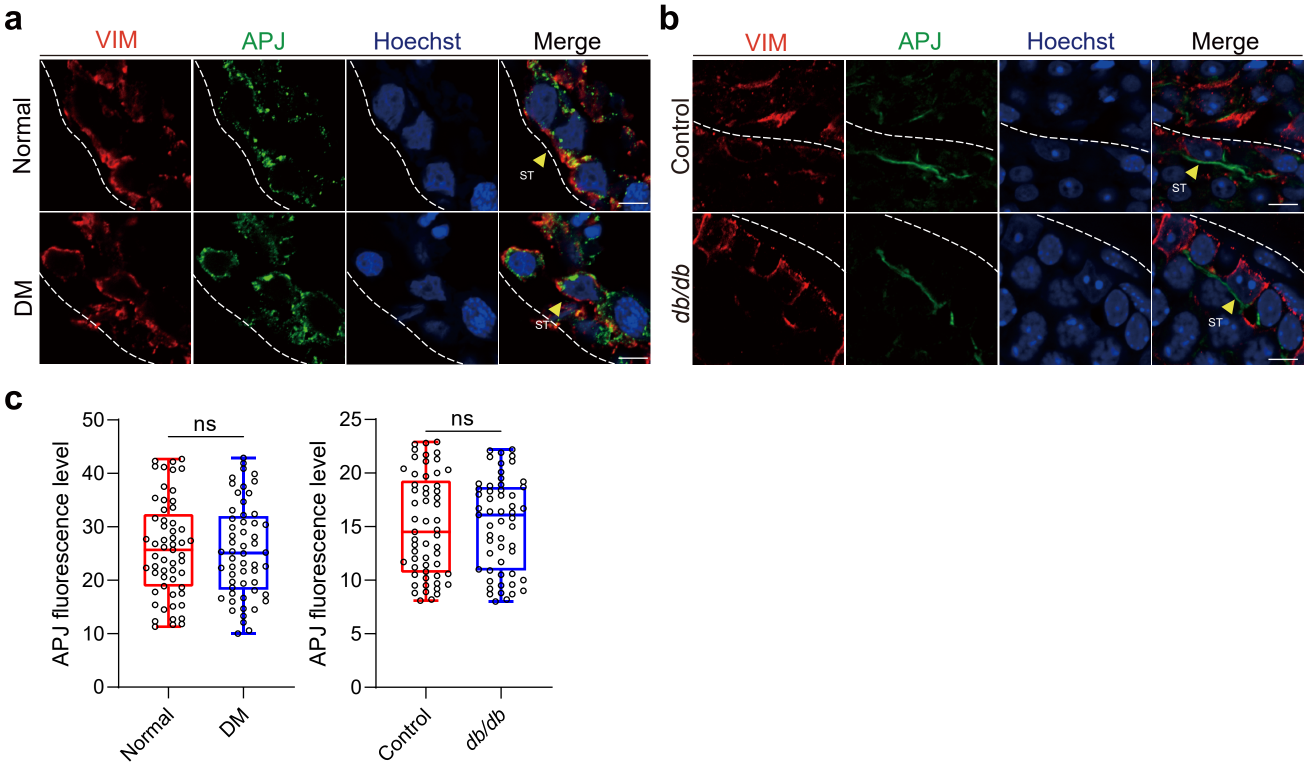
a
VIM
APJ
Hoechst
Merge
Normal
DM
ST
b
VIM
APJ
Hoechst
Merge
Control
db/db
ST
c
APJ fluorescence level
ns
Normal
DM
Control
db/db
0
10
20
30
40
50
5
15
25

Supplementary Figures. 5| APJ expression is not affected in the testis of diabetic mice.

a, Immunofluorescence of VIM (red) co-stained with APJ (green) in normal and diabetic patients' testicular paraffin sections. Scale bar, 10 μm. The yellow arrow indicates the Sertoli cells. **b**, Immunofluorescence of VIM (red) co-stained with APJ (green) in control and *db/db* testicular paraffin sections. Scale bar, 10 μm. The yellow arrow indicates the Sertoli cells. **c**, Quantitative analysis of APJ. ns, not significant. n = 3 per group. Box-and-whisker plots denote the maximum (top whisker), 75th (top edge of box), 25th (bottom edge of box) and minimum (bottom whisker) percentiles, and the median (line in box). Fluorescence intensity values of more than 50 positive cells in at least 5 fields of view were counted in normal and diabetic patients' testicular paraffin sections and *db/db* testicular paraffin sections. Two-tailed student's t test was performed.

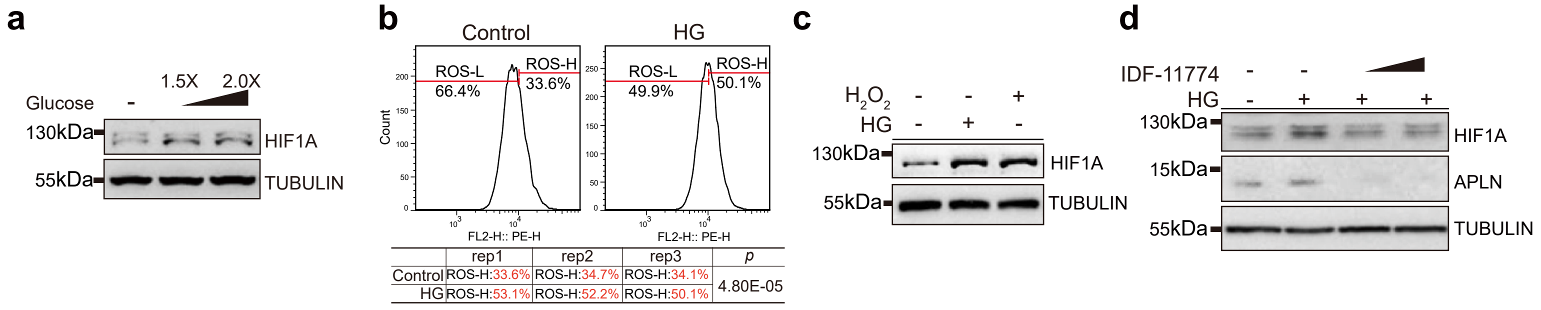
a
Glucose
-
1.5X
2.0X
130kDa
HIF1A
55kDa
TUBULIN
b
Control
HG
ROS-L
66.4%
ROS-H
33.6%
ROS-L
49.9%
ROS-H
50.1%
Count
FL2-H:: PE-H
FL2-H:: PE-H
rep1
rep2
rep3
p
Control ROS-H:33.6% ROS-H:34.7% ROS-H:34.1%
HG ROS-H:53.1% ROS-H:52.2% ROS-H:50.1%
4.80E-05
c
H2O2 - - +
HG - + -
130kDa
HIF1A
55kDa
TUBULIN
d
IDF-11774 - -
HG - + + +
130kDa
HIF1A
15kDa
APLN
55kDa
TUBULIN

Supplementary Figures. 6| High glucose-induced ROS elevation activates HIF1A. **a**, Immunoblots of HIF1A in TM4 treated different concentrations glucose. **b**, Representative figures for TM4 cell treated with high glucose for 24 hours followed by DHE staining and flow cytometry. Results of three independent experiments are shown (n=3). Two-tailed student's t test was performed. **c**, Immunoblots of HIF1A in TM4 treated with high glucose and H_2O_2. **d**, Immunoblots of HIF1A and APLN in TM4 treated different concentrations IDF-11774 (HIF1A inhibitor) and HG.

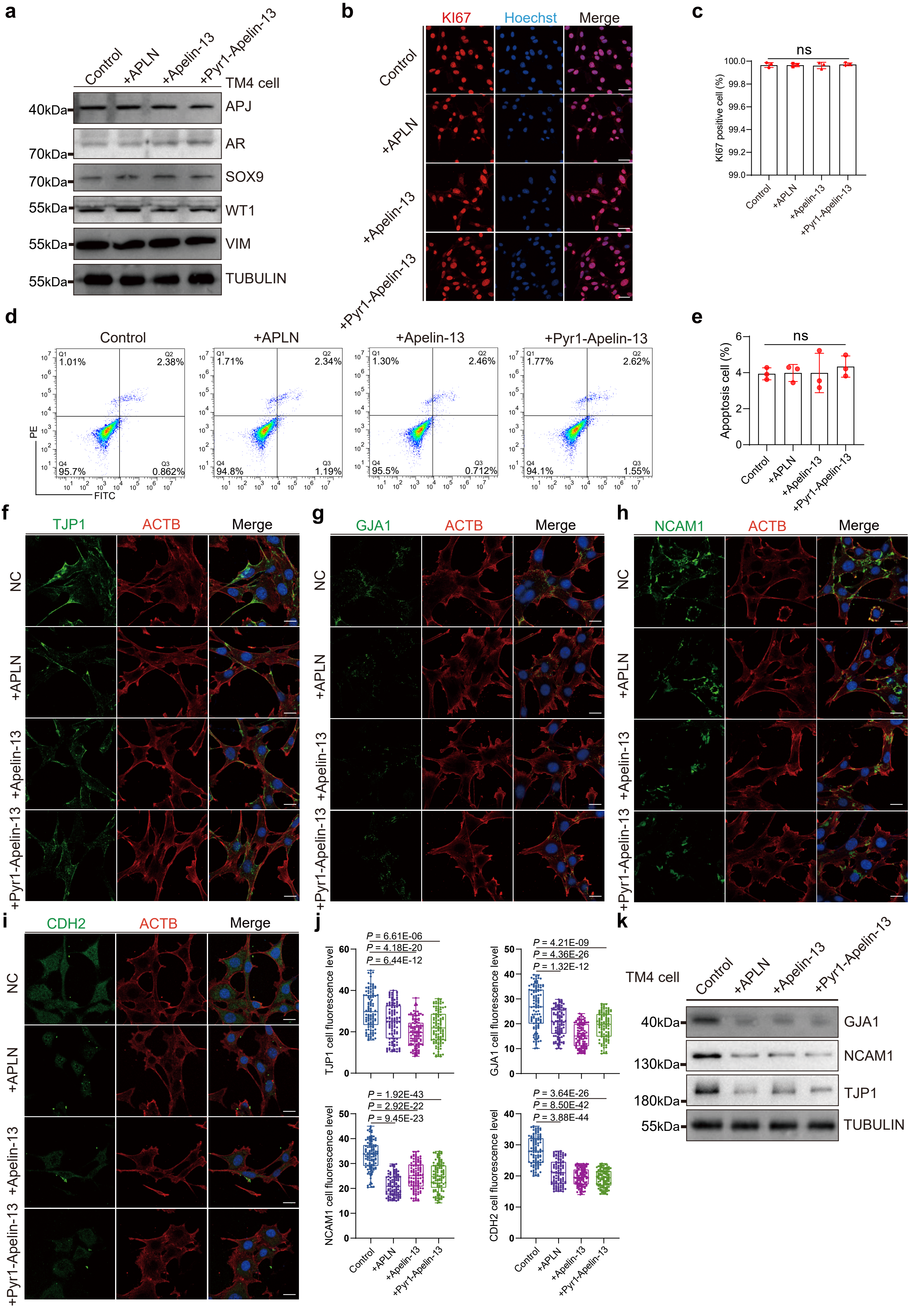
a
Control
+APLN
+Apelin-13
+Pyr1-Apelin-13
TM4 cell
40kDa
APJ
AR
70kDa
70kDa
SOX9
55kDa
WT1
55kDa
VIM
55kDa
TUBULIN
b
KI67
Hoechst
Merge
c
ns
KI67 positive cell (%)
d
Control
+APLN
+Apelin-13
+Pyr1-Apelin-13
PE
FITC
1.01%
2.38%
95.7%
0.862%
1.71%
2.34%
94.8%
1.19%
1.30%
2.46%
95.5%
0.712%
1.77%
2.62%
94.1%
1.55%
e
ns
Apoptosis cell (%)
f
TJP1
ACTB
Merge
NC
g
GJA1
h
NCAM1
i
CDH2
j
P = 6.61E-06
P = 4.18E-20
P = 6.44E-12
TJP1 cell fluorescence level
P = 4.21E-09
P = 4.36E-26
P = 1.32E-12
GJA1 cell fluorescence level
P = 1.92E-43
P = 2.92E-22
P = 9.45E-23
NCAM1 cell fluorescence level
P = 3.64E-26
P = 8.50E-42
P = 3.88E-44
CDH2 cell fluorescence level
k
TM4 cell
40kDa
GJA1
NCAM1
130kDa
TJP1
180kDa
55kDa
TUBULIN

Supplementary Figures. 7| Various active forms of APLN impairs cell junction proteins expression without affecting cell identity and viability. a, Immunoblots of indicated proteins in TM4 treated APLN, Apelin-13 and Pyr1-Apelin-13. **b**, Immunofluorescence of KI67 (red) in TM4 treated APLN, Apelin-13 and Pyr1-Apelin-13. Scale bar, 50 μm. **c**, KI67-positive cells percentage statistics in TM4 treated APLN, Apelin-13 and Pyr1-Apelin-13, Mean ± SEM. ns, not significant, one-way ANOVA. Results of three independent experiments are shown (n=3). **d**, Annexin V-FITC apoptosis assay on TM4 cells treated with APLN, Apelin-13 and Pyr1-Apelin-13. **e**, Apoptosis rate on TM4 cells treated with APLN, Apelin-13 and Pyr1-Apelin-13. Mean ± SEM. ns, not significant, one-way ANOVA. Results of three independent experiments are shown (n=3). **f**, Immunofluorescence of ACTB (red) co-stained with TJP1 (green) on TM4 cells treated with APLN, Apelin-13 and Pyr1-Apelin-13. Scale bar, 10 μm. **g**, Immunofluorescence of ACTB (red) co-stained with GJA1 (green) on TM4 cells treated with APLN, Apelin-13 and Pyr1-Apelin-13. Scale bar, 10 μm. **h**, Immunofluorescence of ACTB (red) co-stained with NCAM1 (green) on TM4 cells treated with APLN, Apelin-13 and Pyr1-Apelin-13. Scale bar, 10 μm. **i**, Immunofluorescence of ACTB (red) co-stained with CDH2 (green) on TM4 cells treated with APLN, Apelin-13 and Pyr1-Apelin-13. Scale bar, 10 μm. **j**, Quantitative analysis of TJP1, GJA1, NCAM1 and CDH2. n = 3 biologically independent samples. Box-and-whisker plots denote the maximum (top whisker), 75th (top edge of box), 25th (bottom edge of box) and minimum (bottom whisker) percentiles, and the median (line in box). Fluorescence intensity values of more than 100 positive cells were counted in TM4 cells treated with APLN, Apelin-13 and Pyr1-Apelin-13. One-way ANOVA was performed. **k**, Immunoblots of GJA1, NCAM1, TJP1 in TM4 treated APLN, Apelin-13 and Pyr1-Apelin-13.

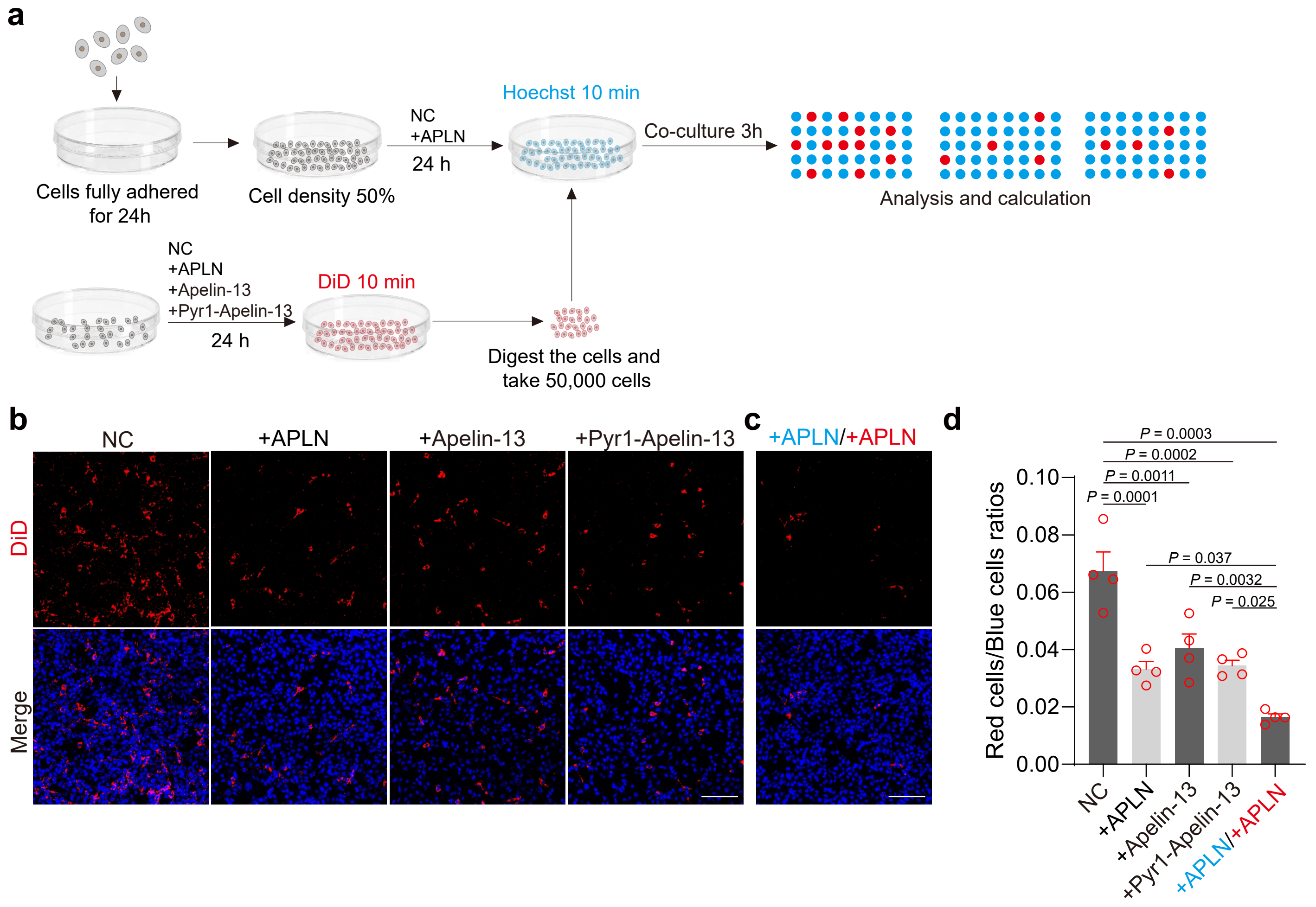
a
Cells fully adhered for 24h
Cell density 50%
NC
+APLN
24 h
Hoechst 10 min
Co-culture 3h
Analysis and calculation
NC
+APLN
+Apelin-13
+Pyr1-Apelin-13
24 h
DiD 10 min
Digest the cells and take 50,000 cells
b
NC
+APLN
+Apelin-13
+Pyr1-Apelin-13
DiD
Merge
c
+APLN/+APLN
d
Red cells/Blue cells ratios
0.10
0.08
0.06
0.04
0.02
0.00
P = 0.0003
P = 0.0002
P = 0.0011
P = 0.0001
P = 0.037
P = 0.0032
P = 0.025
NC
+APLN
+Apelin-13
+Pyr1-Apelin-13
+APLN/+APLN

Supplementary Figures. 8| Cell adhesion assays after treatment of TM4 cells with different APLN and its small molecules. a, Experimental design for cell adhesion assay. **b**, Representative images of DiD (red) immunofluorescence in TM4 cell. Scale bar, 100 μm. **c**, Representative images of DiD (red) immunofluorescence both treated APLN in "red" and "blue" TM4 cell. Scale bar, 100 μm. **d**, Statistics on the ratio of red TM4 cells to blue TM4 cells. n=4 biologically independent experiments. Data are presented as means ± SEM. More than 500 cells were counted in each subgroup. One-way ANOVA was performed.

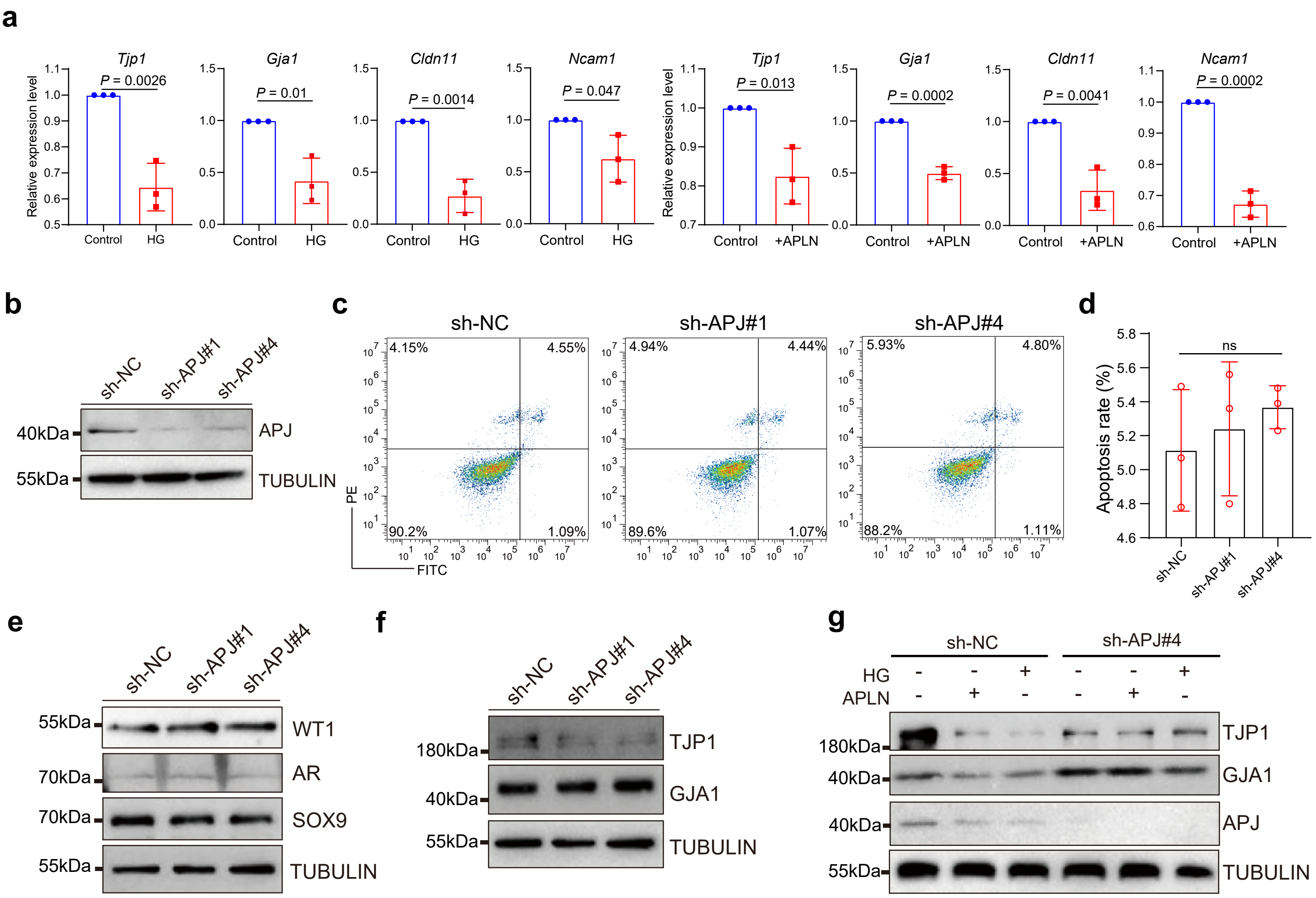
a
Tjp1
P = 0.0026
Gja1
P = 0.01
Cldn11
P = 0.0014
Ncam1
P = 0.047
Relative expression level
Control
HG
Tjp1
P = 0.013
Gja1
P = 0.0002
Cldn11
P = 0.0041
Ncam1
P = 0.0002
Control
+APLN
b
sh-NC
sh-APJ#1
sh-APJ#4
40kDa
APJ
55kDa
TUBULIN
c
sh-NC
sh-APJ#1
sh-APJ#4
4.15%
4.55%
90.2%
1.09%
4.94%
4.44%
89.6%
1.07%
5.93%
4.80%
88.2%
1.11%
PE
FITC
d
Apoptosis rate (%)
ns
sh-NC
sh-APJ#1
sh-APJ#4
e
sh-NC
sh-APJ#1
sh-APJ#4
55kDa
WT1
70kDa
AR
70kDa
SOX9
55kDa
TUBULIN
f
sh-NC
sh-APJ#1
sh-APJ#4
180kDa
TJP1
40kDa
GJA1
55kDa
TUBULIN
g
sh-NC
sh-APJ#4
HG
APLN
180kDa
TJP1
40kDa
GJA1
40kDa
APJ
55kDa
TUBULIN

Supplementary Figures. 9| Targeting APJ restores expression of cell adhesion proteins under high glucose or with APLN treatment. **a**, The relative expression levels of *Ncam1*, *Cldn11*, *Gja1*, *Tjp1* in TM4 cell under high glucose or APLN. Data are presented as means ± SEM. Unpaired two-tailed t test was performed. Results of three independent experiments are shown. n = 3 per group. **b**, Immunoblots of APJ in TM4 cells transfected shRNA of APJ. **c,** Annexin V-FITC apoptosis assay on TM4 cells transfected shRNA of APJ. **d**, Apoptosis rate on TM4 cells transfected shRNA of APJ, ns, not significant, one-way ANOVA. Results of three independent experiments are shown. n = 3 biologically independent cell. Mean ± SEM. **e**, Immunoblots of WT1, AR and SOX9 in stable KD APJ cell. **f**, Immunoblots of TJP1, GJA1 and NCAM1 in stable KD APJ cell. **g**, Immunoblots of indicated proteins in stable KD APJ cell treated APLN or high glucose.

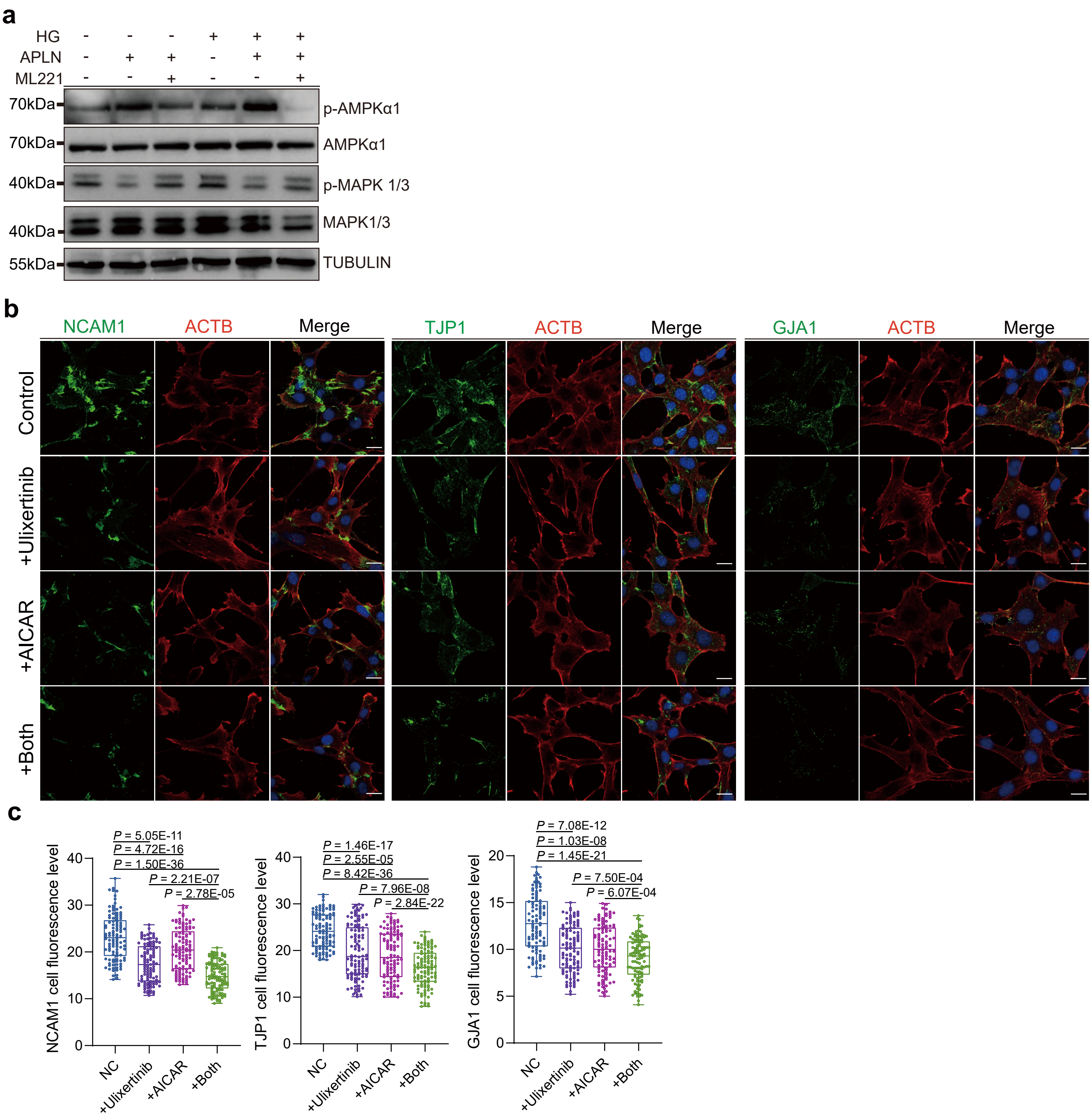
a
HG
APLN
ML221
70kDa
p-AMPKα1
70kDa
AMPKα1
40kDa
p-MAPK 1/3
40kDa
MAPK1/3
55kDa
TUBULIN
b
NCAM1
ACTB
Merge
TJP1
ACTB
Merge
GJA1
ACTB
Merge
Control
+Ulixertinib
+AICAR
+Both
c
P = 5.05E-11
P = 4.72E-16
P = 1.50E-36
P = 2.21E-07
P = 2.78E-05
NCAM1 cell fluorescence level
NC
+Ulixertinib
+AICAR
+Both
P = 1.46E-17
P = 2.55E-05
P = 8.42E-36
P = 7.96E-08
P = 2.84E-22
TJP1 cell fluorescence level
P = 7.08E-12
P = 1.03E-08
P = 1.45E-21
P = 7.50E-04
P = 6.07E-04
GJA1 cell fluorescence level

Supplementary Figures. 10| The AMPK and MAPK pathways are downstream effectors of APLN/APJ in Sertoli cells. a, Immunoblots of AMPKα1, p-AMPKα1, MAPK1/3 and p-MAPK1/3 in TM4 treated high glucose, APLN or both. **b**, Immunofluorescence of ACTB (red) co-stained with TJP1, NCAM1, GJA1 (green) on TM4 cells treated with Ulixertinib, AICAR or both. Scale bar, 10 μm. **c**, Quantitative analysis of TJP1, GJA1, and CDH2. n = 3 per group. Box-and-whisker plots denote the maximum (top whisker), 75th (top edge of box), 25th (bottom edge of box) and minimum (bottom whisker) percentiles, and the median (line in box). Fluorescence intensity values of more than 100 positive cells were counted in TM4 cells treated with Ulixertinib, AICAR or both. Two-tailed student's t test was performed.

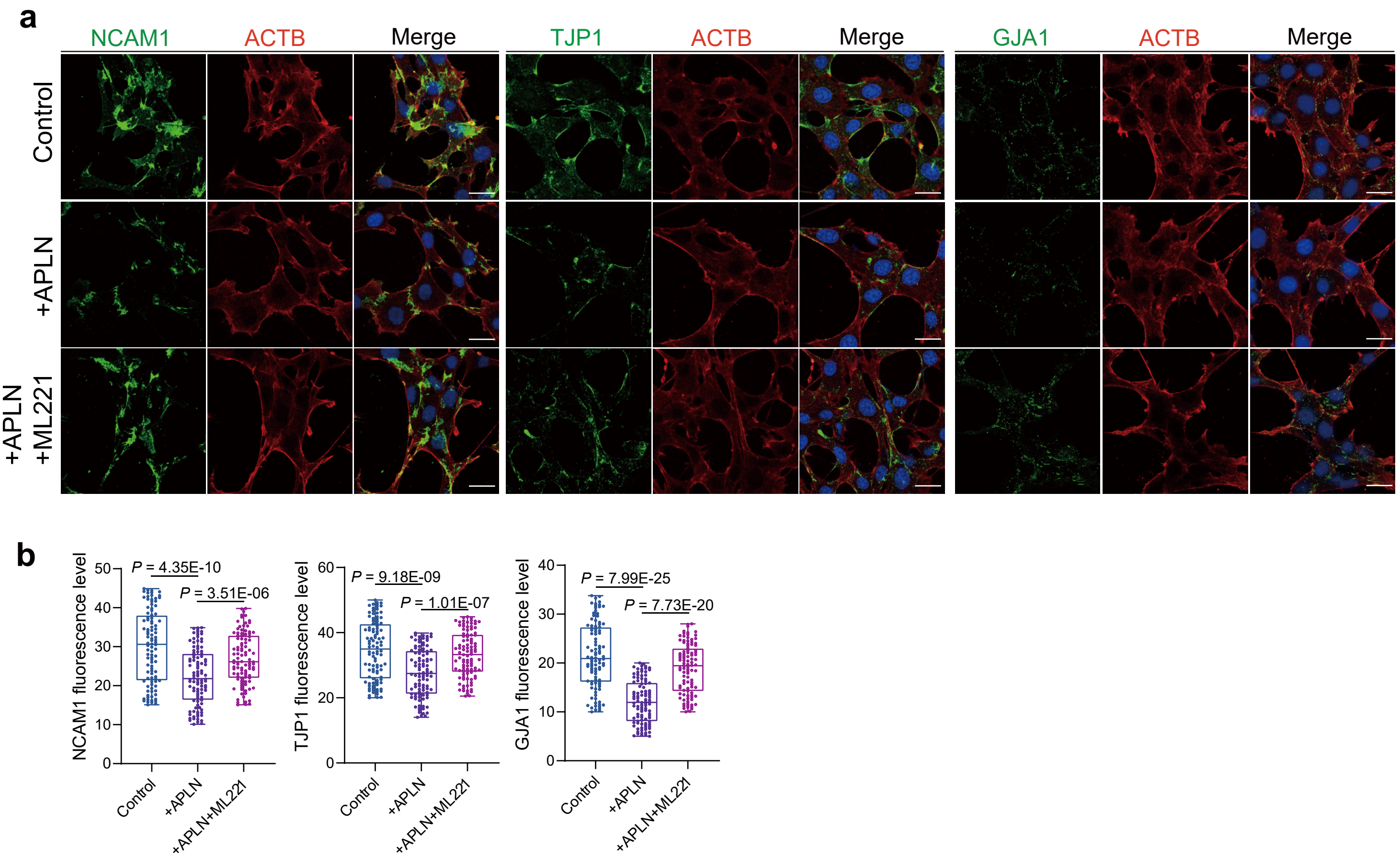
a
NCAM1
ACTB
Merge
TJP1
ACTB
Merge
GJA1
ACTB
Merge
Control
+APLN
+APLN +ML221
b
NCAM1 fluorescence level
P = 4.35E-10
P = 3.51E-06
TJP1 fluorescence level
P = 9.18E-09
P = 1.01E-07
GJA1 fluorescence level
P = 7.99E-25
P = 7.73E-20
Control
+APLN
+APLN+ML221

Supplementary Figures. 11| Compromising APLN effect with ML221 in cultured TM4 cells. a, Immunofluorescence of ACTB (red) co-stained with TJP1, NCAM1, GJA1 (green) on TM4 cells treated with APLN, or APLN combined with ML221. Scale bar, 10 μm. **b**, Quantitative analysis of TJP1, GJA1, and CDH2. n = 3 per group. Box-and-whisker plots denote the maximum (top whisker), 75th (top edge of box), 25th (bottom edge of box) and minimum (bottom whisker) percentiles, and the median (line in box). Fluorescence intensity values of more than 100 positive cells were counted in TM4 cells treated with APLN, or APLN combined ML221. Two-tailed student's t test was performed.

a

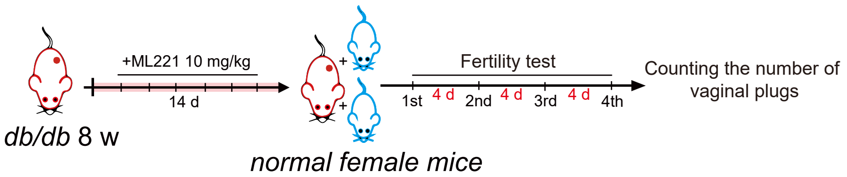

b

Group (n)		No. of estrous female mice	1st vaginal plugs	2nd vaginal plugs	3rd vaginal plugs	4th vaginal plugs
db/db	Control (6)	12	0/12	0/12	0/12	0/12
	ML221 (6)	12	0/12	0/12	0/12	0/12

Supplementary Figures. 12| Mating behavior and fertility test.

a, Schematic illustration of the experimental and analysis workflow. **b**, Fertility testing of control and ML221 group.

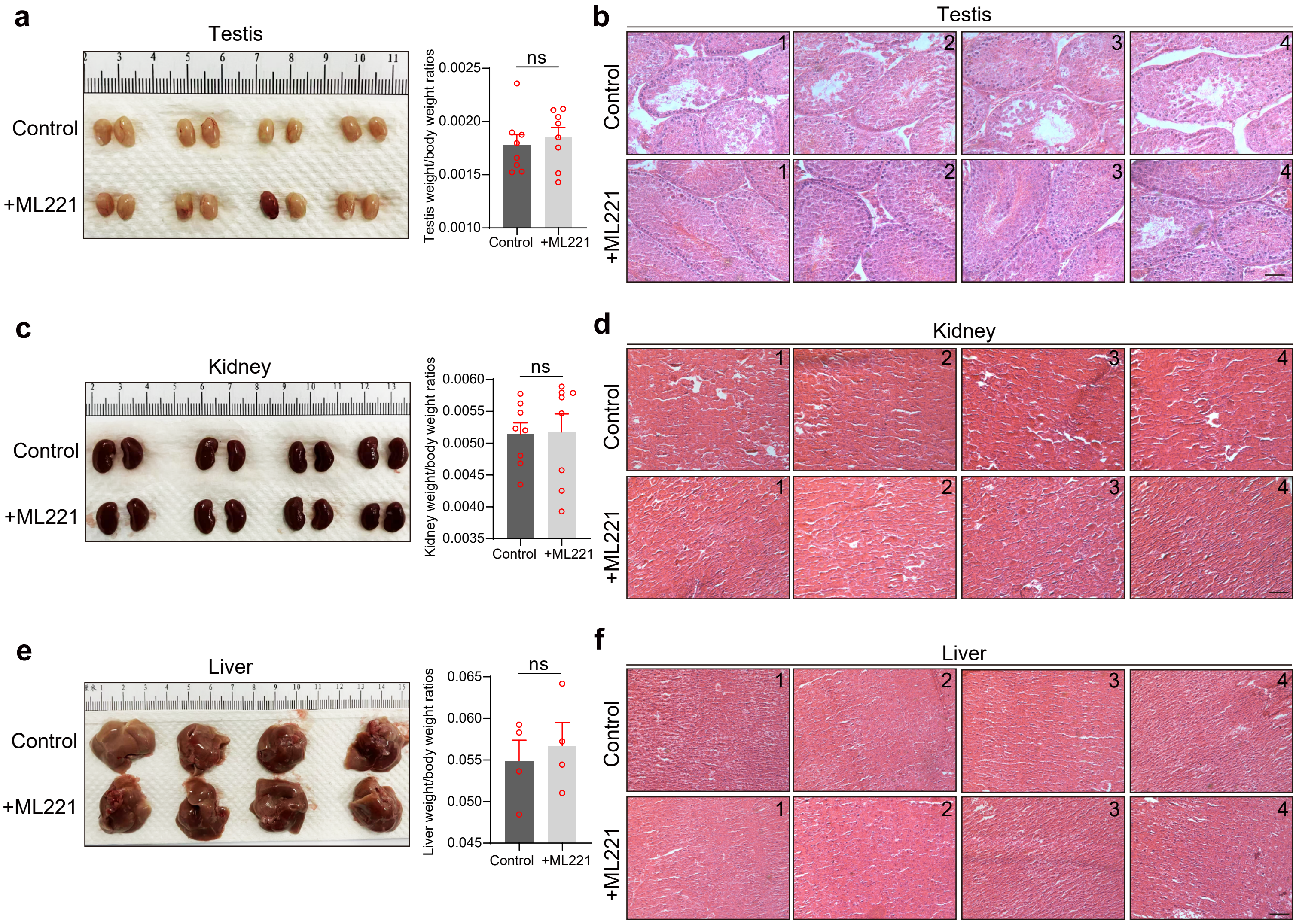
a
Testis
Control
+ML221
Testis weight/body weight ratios
0.0025
0.0020
0.0015
0.0010
ns
Control
+ML221
b
Testis
Control
+ML221
c
Kidney
Control
+ML221
Kidney weight/body weight ratios
0.0060
0.0055
0.0050
0.0045
0.0040
0.0035
ns
d
Kidney
e
Liver
Control
+ML221
Liver weight/body weight ratios
0.065
0.060
0.055
0.050
0.045
ns
f
Liver

Supplementary Figures. 13| Testis, kidney and liver weights and H&E staining between control and ML221 injection group. a, Brightfield diagram of testis between control and ML221 injection group and weight statistics. Each group has four mice. Mean ± SEM. ns, not significant, unpaired two-tailed t test. **b**, H&E staining of testicular sections in control and ML221 injection group. Scale bar, 100 μm. **c**, Brightfield diagram of kidney between control and ML221 injection group and weight statistics. Each group has four mice. Mean ± SEM. ns, not significant, unpaired two-tailed t test. **d**, H&E staining of renal sections in control and ML221 injection group. Scale bar, 200 μm. **e**, Brightfield diagram of liver between control and ML221 injection group and weight statistics. Each group has four mice. ns, not significant, unpaired two-tailed t test. Mean ± SEM. **f**, H&E staining of liver sections in control and ML221 injection group. Scale bar, 200 μm.

a

Mouse numbers		*db/db*-Control 1	2	3	4	*db/db*-ML221 1	2	3	4	C57BL/6N 1	2	3
Sperm counts (million/mL)		1,289	1,187	1,615	1,368	2,956	2,694	4,167	3,011	3,369	3,605	4,016
Sperm motility	PR(%)	7.43	8.15	9.45	10.7	37.1	29.3	26.6	29.9	28.5	42.8	32.9
	NR(%)	4.05	5.73	3.70	0.38	7.46	7.62	7.09	7.73	6.60	5.66	6.86
	IM(%)	88.5	86.1	86.9	85.5	55.5	63.5	66.3	62.2	64.9	51.6	60.28

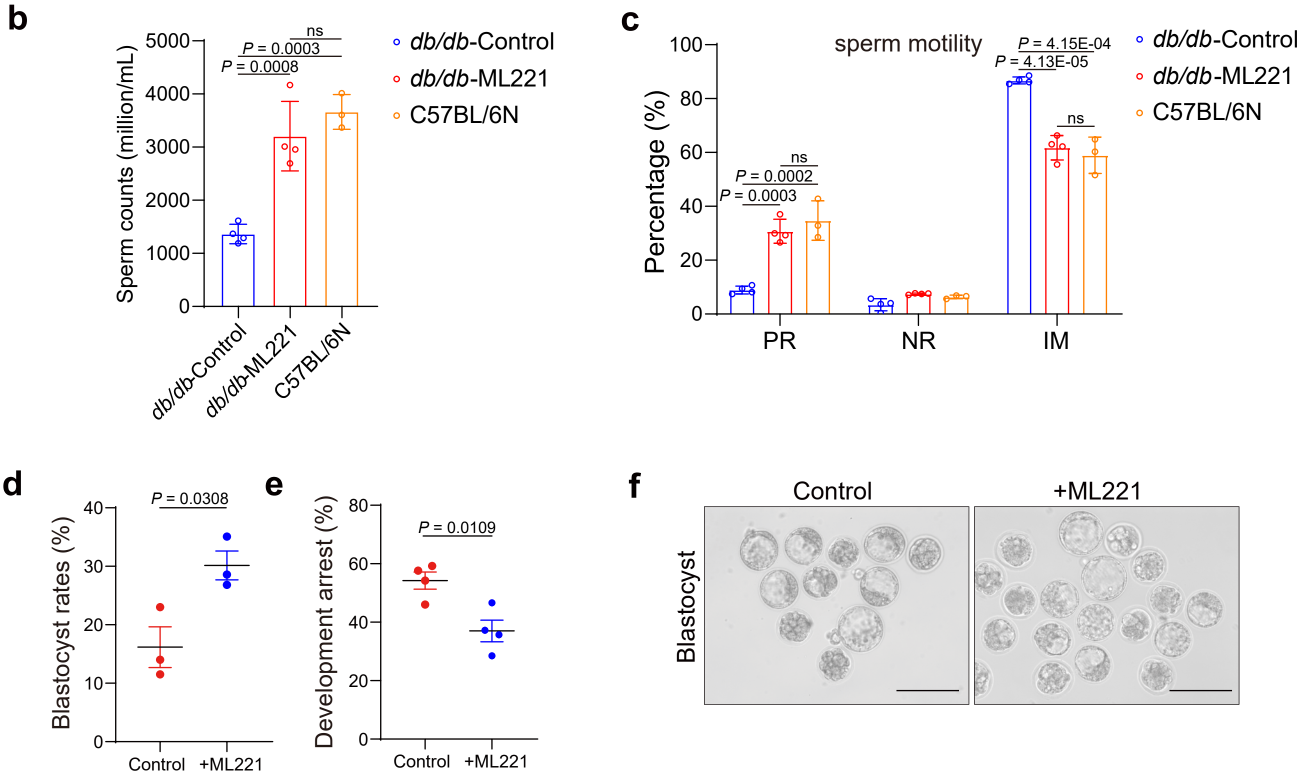

Supplementary Figures. 14| ML221 significantly improved sperm count, motility and ICSI blastocyst rate in *db/db* mice. **a**, Sperm counts and sperm motility level between control, ML221 and C57BL/6N group. PR: progressive motile, NP: non-progressive motile, IM: immotility. **b**, Sperm counts of control, ML221 and C57BL/6N group. ns, not significant, unpaired two-tailed t test. Mean ± SEM. n = 4 mice per group (n = 3 mice for C57BL/6N). **c**, Sperm motility of control, ML221 and C57BL/6N group. Mean ± SEM. ns, not significant, unpaired two-tailed t test. n = 4 mice per group (n = 3 mice for C57BL/6N). **d**, Blastocyst development rate in control and ML221 injection group. n = 3 biologically independent experiments. Mean ± SEM. Unpaired two-tailed t test. **e**, Blastocyst arrest rate in control and ML221 injection group. n = 4 biologically independent experiments. Mean ± SEM. Unpaired two-tailed t test. **f**, Brightfield diagram of blastocyst between control and ML221 injection group. Scale bar, 200 μm.

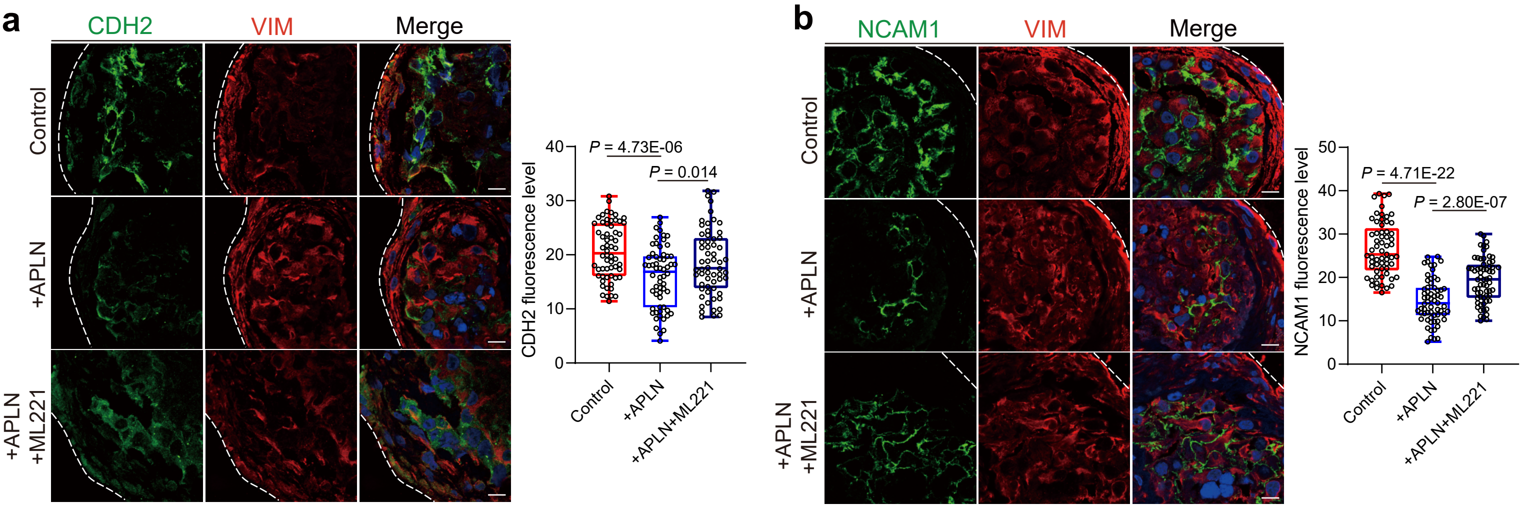

a
CDH2
VIM
Merge
Control
+APLN
+APLN +ML221
P = 4.73E-06
P = 0.014
CDH2 fluorescence level
0
10
20
30
40
Control
+APLN
+APLN+ML221
b
NCAM1
VIM
Merge
Control
+APLN
+APLN +ML221
P = 4.71E-22
P = 2.80E-07
NCAM1 fluorescence level
0
10
20
30
40
50
Control
+APLN
+APLN+ML221

Supplementary Figures. 15| Inhibition APLN improves BTB related gene expression in human cultured testis. a, Immunofluorescence of CDH2 (green) and VIM (red) in human testis culture on Day 7 paraffin sections between indicated sample groups. Scale bar, 20 μm. n = 3 per group. Box-and-whisker plots denote the maximum (top whisker), 75th (top edge of box), 25th (bottom edge of box) and minimum (bottom whisker) percentiles, and the median (line in box). Quantitative analysis of CDH2. Data are presented as means ± SEM. One-way ANOVA was performed. **b,** Immunofluorescence of NCAM1 (green) and VIM (red) in human testis culture on Day 7 paraffin sections between indicated sample groups. Scale bar, 20 μm. n = 3 per group. Box-and-whisker plots denote the maximum (top whisker), 75th (top edge of box), 25th (bottom edge of box) and minimum (bottom whisker) percentiles, and the median (line in box). Quantitative analysis of NCAM1. Unpaired two-tailed t test was performed.

a

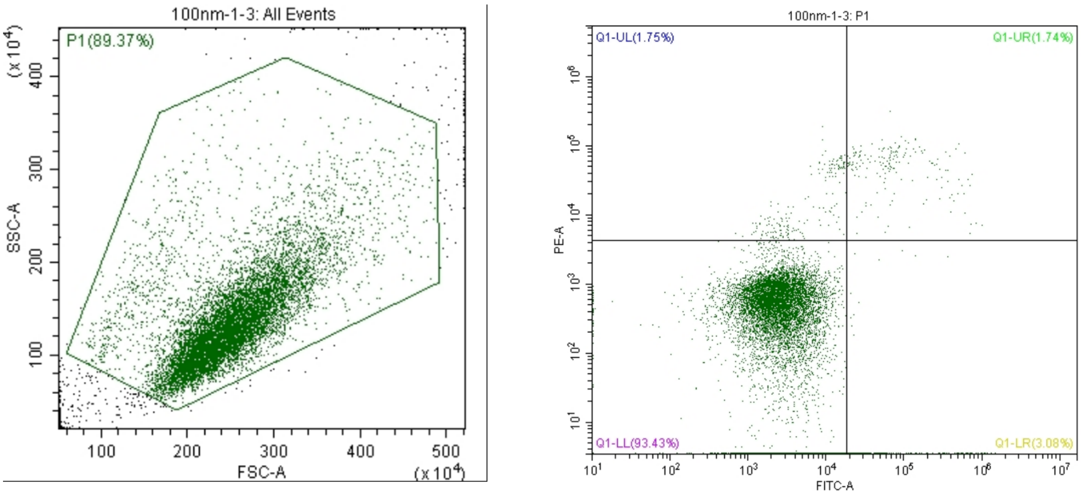

100nm-1-3: All Events
P1(89.37%)
SSC-A
(x10⁴)
FSC-A
(x10⁴)
100nm-1-3: P1
Q1-UL(1.75%)
Q1-UR(1.74%)
Q1-LL(93.43%)
Q1-LR(3.08%)
PE-A
FITC-A

Supplementary Figures. 16| Annexin V-FITC apoptosis assay analysis by FACS.

a, Representative FACS gating scheme for Annexin V-FITC apoptosis assay.
