# Supplementary material for: Targeting APLN/APJ restores blood-testis barrier and improves spermatogenesis in murine and human diabetic models
Source: Nat Commun. 2022 Nov 28;13:7335. doi: 10.1038/s41467-022-34990-3 (PMC9705293; doi:10.1038/s41467-022-34990-3)
Supplement: Supplementary file 2 — Description of Additional Supplementary Files [file 41467_2022_34990_MOESM2_ESM.pdf]

Description of Additional Supplementary Files

File Name: Supplementary Data 1
Description: Total differential genes and metabolism-related genes related to Fig. 1d. P value was calculated by wilcoxon rank sum test.

File Name: Supplementary Data 2
Description: DEGs and GSEA result of Sertoli cells related to Fig. 2. *P* value was calculated by permutation test.
