# Supplementary material for: Targeting APLN/APJ restores blood-testis barrier and improves spermatogenesis in murine and human diabetic models
Source: Nat Commun. 2022 Nov 28;13:7335. doi: 10.1038/s41467-022-34990-3 (PMC9705293; doi:10.1038/s41467-022-34990-3)
Supplement: Supplementary file 5 — Reporting Summary [file 41467_2022_34990_MOESM5_ESM.pdf]

Corresponding author(s): Xiao-Yang Zhao

Last updated by author(s): Oct 29, 2022

Reporting Summary

Nature Portfolio wishes to improve the reproducibility of the work that we publish. This form provides structure for consistency and transparency in reporting. For further information on Nature Portfolio policies, see our Editorial Policies and the Editorial Policy Checklist.

Statistics

For all statistical analyses, confirm that the following items are present in the figure legend, table legend, main text, or Methods section.

n/a	Confirmed	
☐	☒	The exact sample size (n) for each experimental group/condition, given as a discrete number and unit of measurement
☐	☒	A statement on whether measurements were taken from distinct samples or whether the same sample was measured repeatedly
☐	☒	The statistical test(s) used AND whether they are one- or two-sided<br>*Only common tests should be described solely by name; describe more complex techniques in the Methods section.*
☒	☐	A description of all covariates tested
☒	☐	A description of any assumptions or corrections, such as tests of normality and adjustment for multiple comparisons
☐	☒	A full description of the statistical parameters including central tendency (e.g. means) or other basic estimates (e.g. regression coefficient) AND variation (e.g. standard deviation) or associated estimates of uncertainty (e.g. confidence intervals)
☐	☒	For null hypothesis testing, the test statistic (e.g. F, t, r) with confidence intervals, effect sizes, degrees of freedom and P value noted<br>*Give P values as exact values whenever suitable.*
☒	☐	For Bayesian analysis, information on the choice of priors and Markov chain Monte Carlo settings
☐	☒	For hierarchical and complex designs, identification of the appropriate level for tests and full reporting of outcomes
☐	☒	Estimates of effect sizes (e.g. Cohen's d, Pearson's r), indicating how they were calculated

Our web collection on statistics for biologists contains articles on many of the points above.

Software and code

Policy information about availability of computer code

Data collection	The scRNA-seq, RNA-seq and CUT-Tag was performed on an Illumina Novaseq platform.<br>Quantitative fluorescence analysis with imageJ.<br>Flow cytometric assays were performed on CytoFLEX and analysed in FlowJo
Data analysis	R packages: Seurat (v3.0.0); Monocle3 (v0.1.3); clusterProfiler (v3.10.1); CellCall (v0.0.0.9); nichenetr (v0.1.0); ggplot2 (v3.1.1).<br>Main softwares: Hisat2 (v2.1.0); HTSeq (v0.11.3); bbduk (v38.18); trimmomatic (v0.39); bowtie2 (v2.3.5.1); macs2 (v2.2.6); bedGraphToBigWig (v2.8); R (v3.5.3).<br>GraphPad Prism 9.2.0, FlowJo 10.0.8, imageJ 1.8.0, CytExpert 2.4.0<br>The code used in this paper is deposited at https://github.com/YangXinyan/DM-scRNA-seq (https://doi.org/10.5281/zenodo.7261195).

For manuscripts utilizing custom algorithms or software that are central to the research but not yet described in published literature, software must be made available to editors and reviewers. We strongly encourage code deposition in a community repository (e.g. GitHub). See the Nature Portfolio guidelines for submitting code & software for further information.

Data

Policy information about availability of data

All manuscripts must include a data availability statement. This statement should provide the following information, where applicable:

- Accession codes, unique identifiers, or web links for publicly available datasets
- A description of any restrictions on data availability
- For clinical datasets or third party data, please ensure that the statement adheres to our policy

Raw sequencing data for human normal testis was retrieved from Gene Expression Omnibus (GEO) under accession number GSE106487 (https://www.ncbi.nlm.nih.gov/geo/query/acc.cgi?acc=GSE106487). The single-cell RNA sequencing raw data from human diabetic patients' testis in this paper have been deposited in the Genome Sequence Archive in National Genomics Data Center, China National Center for Bioinformation / Beijing Institute of Genomics, Chinese Academy of Sciences (GSA-Human: HRA000976) that are publicly accessible at https://ngdc.cncb.ac.cn/gsa-human/browse/HRA000976. The HIF1A CUT&Tag raw data in this paper have been deposited in the Genome Sequence Archive (GSA: CRA004696) that are publicly accessible at https://ngdc.cncb.ac.cn/gsa/browse/CRA004696. The processed data in this paper have been deposited in the Gene Expression Omnibus (GEO) at NCBI under accession number GSE179080 (https://www.ncbi.nlm.nih.gov/geo/query/acc.cgi?acc=GSE179080).

Field-specific reporting

Please select the one below that is the best fit for your research. If you are not sure, read the appropriate sections before making your selection.

☒ Life sciences ☐ Behavioural & social sciences ☐ Ecological, evolutionary & environmental sciences

For a reference copy of the document with all sections, see nature.com/documents/nr-reporting-summary-flat.pdf

Life sciences study design

All studies must disclose on these points even when the disclosure is negative.

Sample size	For scRNAseq: we obtained intact seminiferous epithelium from two T2DM patients with ethics approval via testicular sperm aspiration (TESA). These two diabetic patients had complete spermatogenesis, and all cell types could be captured completely using single cell sequencing technology.<br>For immunofluorescence and seminiferous tubule in vitro culture: we obtained intact seminiferous epithelium from three obstructive azoospermia males diagnosed with diabetes and three obstructive azoospermia males with nomal spermatogenesis undergoing sperm isolation surgery for in vitro fertilization with ethics approval.<br>Quantification of immunofluorescence: fluorescence intensity values of more than 50 positive cells in at least 5 fields of view were counted in normal and diabetic patients' testicular paraffin sections.<br>Blood testis barrier intergrity assay: C57BL/6N or db/db mice were randomly divided into three groups. Statistics were performed in n from a minimum of 4 to a maximum of 6 mouse testes each group.
Data exclusions	We exclude the cells that failed quality control and explicitly define all filter criteria in the Methods.
Replication	At least three independent biological replicates were performed for each development stages in spermatogenesis.
Randomization	Single cells of collected samples were randomly picked with mouth pipette, and library preparation and sequencing was randomized to avoid batch effects. All single cells were allocated into different groups according to their development stages in spermatogenesis.<br>For blood testis barrier intergrity assay, C57BL/6N or db/db mice were randomly divided into three groups.<br>Quantification of immunofluorescence is grouped statistics according to the treatment.
Blinding	Investigators were blinded to group allocation during analysis of staining

Reporting for specific materials, systems and methods

We require information from authors about some types of materials, experimental systems and methods used in many studies. Here, indicate whether each material, system or method listed is relevant to your study. If you are not sure if a list item applies to your research, read the appropriate section before selecting a response.

Materials & experimental systems

n/a	Involved in the study
☐	☒ Antibodies
☐	☒ Eukaryotic cell lines
☒	☐ Palaeontology and archaeology
☐	☒ Animals and other organisms
☐	☒ Human research participants
☒	☐ Clinical data
☒	☐ Dual use research of concern

Methods

n/a	Involved in the study
☐	☒ ChIP-seq
☐	☒ Flow cytometry
☒	☐ MRI-based neuroimaging

Antibodies

Antibodies used

For immunofluorescence, commercial primary antibodies were used:
rabbit-anti-CX43 (1:200, Abcam, ab217676, EPR21153,),
rabbit-anti-ZO-1 (1:400, Abcam, ab221547, EPR19945-296, RRID:AB_2892660),
rabbit-anti-CDH2 (1:100, Abcam, ab18203, RRID:AB_444317),
rabbit-anti-NCAM1 (1:100, Abcam, ab220360, EPR21827),
rabbit-anti-HIF1A (1:50, Proteintech, 20960-1-AP, RRID:AB_10732601),
mouse-anti-ACTB (1:400, Proteintech, 66009-1-Ig, RRID:AB_2687938),
mouse-anti-VIMENTIN (1:50, Proteintech, 60330-1-Ig, RRID:AB_2881439),
mouse anti-DDX4 (1:500, Abcam, ab27591, mAbcam27591, RRID:AB_11139638),
rabbit anti-SOX9 (1:200, Millipore, AB5535, RRID:AB_2239761),
rabbit anti-SYCP3 (1:400, Abcam, ab15093, RRID:AB_301639),
mouse anti-CREM (1:50, Santa Cruz, sc-390426),
rabbit anti-KIT (1:200, Abcam, ab32363, YR145, RRID:AB_731513),
mouse anti-FGFR3 (1:30, Santa Cruz, sc-13121, RRID:AB_627596),
rabbit anti-STRA8 (1:100, Millipore, ABN1656),
mouse anti-γH2AX (1:400, Abcam, ab26350, 9F3, RRID:AB_470861),
rabbit anti-INSL3 (1:300, Novus Biologicals, NBP1-81223, RRID:AB_11030510).
For WB, commercial primary antibodies were used:
rabbit-anti-CX43 (1:1000, Proteintech, 26980-1-AP, RRID:AB_2880711),
rabbit-anti-ZO-1 (1:1000, Proteintech, 21773-1-AP, RRID: AB_10733242),
rabbit-anti-APJ (1:1000, Proteintech, 20341-1-AP, RRID: AB_2878676),
rabbit-anti-ERK1/2 (1:1000, CST, 4695T, RRID:AB_390779),
rabbit-anti-pERK1/2 (1:1000, CST, 4370T, RRID:AB_2315112),
rabbit-anti-AMPKα1 (1:1000, CST, 5831T, RRID:AB_10622186),
rabbit-anti- p-AMPKα1 (1:1000, CST, 2535T, 40H9, RRID:AB_331250),
rabbit-anti-SOX11 (1:1000, Abcam, ab134107, EPR8192, RRID:AB_2721126),
mouse anti-SOX9 (1:1000, Abcam, ab76997, 3C10, RRID:AB_2194156),
rabbit-anti-HIF1A (1:1000, Proteintech, 20960-1-AP, RRID:AB_10732601),
rabbit-anti-WT1 (1:1000, Proteintech, 12609-1-AP, RRID:AB_2216225),
rabbit-anti-AR (1:1000, Proteintech, 22089-1-AP, RRID:AB_11182176),
rabbit-anti-VIMENTIN (1:1000, Proteintech, 10366-1-AP, RRID:AB_2273020),
rabbit-anti-HIF1α (1:1000, Proteintech, 20960-1-AP)
mouse anti-TUBULIN (1:10000, SUNGENE, KM9007).

Validation

Validation information was provided by manufacturer.
https://www.abcam.cn/connexin-43--gja1-antibody-epr21153-ab217676.html
https://www.abcam.cn/zo1-tight-junction-protein-antibody-epr19945-296-ab221547.html
https://www.abcam.cn/n-cadherin-antibody-intercellular-junction-marker-ab18203.html
https://www.abcam.cn/ncam1-antibody-epr21827-ab220360.html
https://www.ptgcn.com/products/HIF1A-Antibody-20960-1-AP.htm
https://www.ptgcn.com/products/Pan-Actin-Antibody-66009-1-Ig.htm
https://www.ptgcn.com/products/Vimentin-Antibody-60330-1-Ig.htm
https://www.abcam.cn/ddx4--mvh-antibody-mabcam27591-ab27591.html
https://www.merckmillipore.com/CN/zh/product/Anti-Sox9-Antibody,MM_NF-AB5535
https://www.abcam.cn/scp3-antibody-ab15093.html
https://www.scbt.com/p/crem-antibody-c-2?requestFrom=search
https://www.abcam.cn/c-kit-antibody-yr145-ab32363.html
https://www.scbt.com/p/fgfr-3-antibody-b-9?requestFrom=search
https://www.merckmillipore.com/CN/zh/product/Anti-Stra8-Antibody,MM_NF-ABN1656
https://www.abcam.cn/gamma-h2ax-phospho-s139-antibody-9f3-ab26350.html
https://www.novusbio.com/products/insl3-antibody_nbp1-81223
WB
https://www.ptgcn.com/products/Connexin-43-Antibody-26980-1-AP.htm
https://www.ptgcn.com/products/ZO1-Antibody-21773-1-AP.htm
https://www.ptgcn.com/products/APLNR-Antibody-20341-1-AP.htm
https://www.cellsignal.cn/products/primary-antibodies/p44-42-mapk-erk1-2-137f5-rabbit-mab/4695?site-search-type=Products&N=4294956287&Ntt=4695t&fromPage=plp&_requestid=6020973
https://www.cellsignal.cn/products/primary-antibodies/phospho-p44-42-mapk-erk1-2-thr202-tyr204-d13-14-4e-xp-rabbit-

mab/4370?site-search-type=Products&N=4294956287&Ntt=4370t&fromPage=plp&_requestid=6021634
https://www.cellsignal.cn/products/primary-antibodies/ampka-d5a2-rabbit-mab/5831?site-search-type=Products&N=4294956287&Ntt=5831t&fromPage=plp&_requestid=6021832
https://www.cellsignal.cn/products/primary-antibodies/phospho-ampka-thr172-40h9-rabbit-mab/2535?site-search-type=Products&N=4294956287&Ntt=2535t&fromPage=plp&_requestid=6022164
https://www.abcam.cn/sox11-antibody-epr8192-ab134107.html
https://www.abcam.cn/sox9-antibody-3c10-bsa-and-azide-free-ab76997.html
https://www.ptgcn.com/products/HIF1A-Antibody-20960-1-AP.htm
https://www.ptgcn.com/products/WT1-Antibody-12609-1-AP.htm
https://www.ptgcn.com/products/AR-Antibody-22089-1-AP.htm
https://www.ptgcn.com/products/VIM-Antibody-10366-1-AP.htm
http://www.sungenebiotech.com/index.php?m=Product&a=product_xq&catid=2&proid=53&prid=291&pid=723&id=1566

Eukaryotic cell lines

Policy information about cell lines

Cell line source(s): The TM4 and HEK293T cell line (immortalized mouse Sertoli cells) was purchased from Procell Life Science Technology

Authentication: Cell identity determined correctly by STR assay

Mycoplasma contamination: All cell lines were test negative for Mycoplasma contamination.

Commonly misidentified lines (See ICLAC register): No commonly misidentified lines were used.

Animals and other organisms

Policy information about studies involving animals; ARRIVE guidelines recommended for reporting animal research

Laboratory animals: 4 week male C57BL/6N mice purchased from Southern Medical University Laboratory Animal Center and 8 week male db/db mice were purchased from Cavens Experimental Animal Co., Ltd

Wild animals: This study didn't involve wild animals.

Field-collected samples: This study didn't involve field-collected samples.

Ethics oversight: All animal studies were performed in accordance with the ethical guidelines of South Medical University ethics committee (L2016149).

Note that full information on the approval of the study protocol must also be provided in the manuscript.

Human research participants

Policy information about studies involving human research participants

Population characteristics: Adult human testicular samples were obtained from obstructive azoospermia males diagnosed with T2DM undergoing sperm isolation surgery and obstructive azoospermia males with nomal spermatogenesis undergoing sperm isolation surgery for in vitro fertilization.

Recruitment: Adult human testicular samples for single-cell RNA sequencing analysis were obtained from two obstructive azoospermia males diagnosed with diabetes (DM1: 42 years old; DM2: 34 years old) undergoing sperm isolation surgery for in vitro fertilization. Adult human testicular samples for immunofluorescence and seminiferous tubule in vitro culture were obtained from three obstructive azoospermia males diagnosed with T2DM and three obstructive azoospermia males with nomal spermatogenesis undergoing sperm isolation surgery for in vitro fertilization. All patients signed informed consent forms and voluntarily donated testicular tissue for this study. There was no selection bias.

Ethics oversight: The experiments performed in this study were approved by Third Affiliated Hospital of Guangzhou Medical University (2017-055). The study was performed according to the guidelines of the ethics committee at the third Affiliated Hospital of Guangzhou Medical University. The study design and conduct complied with all relevant regulations regarding the use of human study participants and was conducted in accordance with the criteria set by the Declaration of Helsinki.

Note that full information on the approval of the study protocol must also be provided in the manuscript.

ChIP-seq

Data deposition

☒ Confirm that both raw and final processed data have been deposited in a public database such as GEO.

☒ Confirm that you have deposited or provided access to graph files (e.g. BED files) for the called peaks.

Data access links<br>*May remain private before publication.*	The accession number for the HIF1α CUT&Tag raw data in this paper is GSA: CRA004696 (https://ngdc.cncb.ac.cn/gsa/browse/CRA004696).<br><br>The accession number for the processed data in this paper is GEO: GSE179080 (https://www.ncbi.nlm.nih.gov/geo/query/acc.cgi?acc=GSE179080).
Files in database submission	HIF1A.NC.rep1_1.fq.gz<br>HIF1A.NC.rep1_2.fq.gz<br>HIF1A.NC.rep2_1.fq.gz<br>HIF1A.NC.rep2_2.fq.gz<br>HIF1A.HG.rep1_1.fq.gz<br>HIF1A.HG.rep1_2.fq.gz<br>HIF1A.HG.rep2_1.fq.gz<br>HIF1A.HG.rep2_2.fq.gz<br>HIF1A.NC.rep1.bw<br>HIF1A.NC.rep2.bw<br>HIF1A.HG.rep1.bw<br>HIF1A.HG.rep2.bw<br>HIF1A.NC.rep1.narrowPeak<br>HIF1A.NC.rep2.narrowPeak<br>HIF1A.HG.rep1.narrowPeak<br>HIF1A.HG.rep2.narrowPeak
Genome browser session<br>(e.g. UCSC)	The link for reviewers to access the data is available at: https://www.ncbi.nlm.nih.gov/geo/query/acc.cgi?acc=GSE179080.

Methodology

Replicates	Two independent biological replicates were performed in each group.
Sequencing depth	HIF1A.NC.rep1: 35064727(total number of reads), 25315406(uniquely mapped reads), 150bp paired-end.<br>HIF1A.NC.rep2: 28192017(total number of reads), 21617276(uniquely mapped reads), 150bp paired-end.<br>HIF1A.HG.rep1: 28503774(total number of reads), 21929364(uniquely mapped reads), 150bp paired-end.<br>HIF1A.HG.rep2: 26492849(total number of reads), 20663545(uniquely mapped reads), 150bp paired-end.
Antibodies	rabbit-anti-HIF1α (1:50, Proteintech, 20960-1-AP, Lot:00096129)
Peak calling parameters	mapping: bowtie2 -q --phred33 --very-sensitive --end-to-end -p 2 --reorder -x Mus_musculus.GRCm38.Ensembl.genome -1 Read1.fq.gz -2 Read2.fq.gz -S sample.sam<br>peak calling: macs2 callpeak --verbose 3 -t sample.bam -n sample -g mm -f BAMPE --cutoff-analysis -B --call-summits
Data quality	The q-value (minimum FDR) cutoff 0.05 is used to call significant regions. The four samples of HIF1A.NC.rep1, HIF1A.NC.rep2, HIF1A.HG.rep1, HIF1A.HG.rep2 have 64345, 62154, 77906, 74286 peak which FDR values less than 0.05, respectively. And they have 16492, 16436 , 22921, 22348 peak fold-change is greater than 5, respectively.
Software	Main softwares: bbduk (v38.18); trimmomatic (v0.39); bowtie2 (v2.3.5.1); macs2 (v2.2.6); bedGraphToBigWig (v2.8).

Flow Cytometry

Plots

Confirm that:

☒ The axis labels state the marker and fluorochrome used (e.g. CD4-FITC).

☒ The axis scales are clearly visible. Include numbers along axes only for bottom left plot of group (a 'group' is an analysis of identical markers).

☒ All plots are contour plots with outliers or pseudocolor plots.

☒ A numerical value for number of cells or percentage (with statistics) is provided.

Methodology

Sample preparation	We used Annexin V-FITC/PI to detect cell apoptosis. Cells from different treatments were digested and resuspended followed by three times washes using PBS then then stained with Annexin V- FITC/PI (Vazyme, A211-01) for 15 minutes at 37°C in

	Binding buffer. After adding 4 times the volume of Binding buffer, the flow cytometry can be performed directly. For cell ROS Measuring, TM4 cell were incubated with 2.5 μmol/L DHE (Macklin, D807594) for 25 min in the dark at 37°C and washed two time with PBS and then red fluorescence was detected by flow cytometry.
Instrument	All flow cytometric assays were performed on CytoFLEX (Beckman)
Software	CytExpert 1.2.11.0 (Beckman) was used to analysis FACS date
Cell population abundance	The FACS experiments in this paper do not involve cell purity
Gating strategy	Cell debris was first discarded on the basis of FSC-A and SSC. For apoptosis, we consider FITC+/ PI+ cells as late apoptotic cells and FITC+/PI- as early apoptotic cells. For cell ROS Measuring, when the FL2-H::PE-H channel intensity is greater than 10^4, we determine that it is high ROS.

☒ Tick this box to confirm that a figure exemplifying the gating strategy is provided in the Supplementary Information.
